# Checklist of tortoise beetles (Coleoptera, Chrysomelidae, Cassidinae) from Colombia with new data and description of a new species

**DOI:** 10.3897/zookeys.518.9350

**Published:** 2015-08-25

**Authors:** Lech Borowiec, Jolanta Świętojańska

**Affiliations:** 1 Department of Biodiversity and Evolutionary Taxonomy, University of Wrocław, Przybyszewskiego 63/77, 51-148 Wrocław, Poland

**Keywords:** Coleoptera, Chrysomelidae, Cassidinae, *Cyrtonota abrili*, new faunistic data, checklist, Colombia

## Abstract

A new tortoise beetle species, *Cyrtonota abrili*, is described from the Antioquia and Caldas departments in Colombia. New faunistic data are provided for 87 species, including 16 new additions to the country’s fauna. A checklist of the known 238 species of tortoise beetles recorded from Colombia is given.

## Introduction

Colombian tortoise beetles [Coleoptera, Chrysomelidae, Cassidinae, in the new sense excl. the tribes Cephaloleiini (= Imatidiini) and Spilophorini] (Borowiec 1995, Staines 2002, Sekerka 2014) are poorly known because most data are devoted to original descriptions of new taxa and no regional catalogues or checklists have been published (Borowiec and Świętojańska 2014b). Although 221 species were recorded from the country hitherto, most of them have been noted without precise location or even province name (Borowiec 1999). The most recent data of several dozen species were published by Borowiec (1996, 2002a, 2009b) based on materials from various museums and private collections but outside Colombian institutions. Recently, we had the opportunity to study specimens preserved in the Museo Entomológico UNAB, Universidad Nacional de Colombia, Bogotá. The collection includes specimens representing 87 species of tortoise beetles, including one species new to science and 16 species new to the country. The material was collected in the last fifty years and most specimens are well-labeled with department and locality data, including geographical coordinates. In this paper we describe this new species of *Cyrtonota* Chevrolat, 1837, after reviewing the material preserved in the Museo Entomológico UNAB, Universidad Nacional de Colombia, Bogotá, and provide a checklist of 238 species of tortoise beetles known from Colombia, including department data for each taxon, when available.

## Methods

Taxa in the faunistic list below are arranged alphabetically, by tribe, genus and species. Geographical coordinates are in DMS format.The new species was identified to the genus *Cyrtonota* based on previous studies (Borowiec 2007a, 2009a; Sekerka 2007) that examined all the other known species.

Photos were taken using Nikon SMZ 1500 stereomicroscope with Nikon Coolpix 4500 photo camera as several separate layers and combined in the Helicon Focus software. Exact label data are cited for type material. A forward slash (/) separates different lines.

## Description of a new species

### Cyrtonota abrili

Taxon classificationAnimaliaColeopteraChrysomelidae

Borowiec & Świętojańska sp. n.

http://zoobank.org/84D93316-606F-4FC4-9E7B-2A307D0C5B65

#### Type locality.

Colombia, Antioquia department, Envigado city, El Salado quarter, 1573 m a.s.l.

#### Type material.

Holotype: “Colombia, Antioquia, / Envigado El Salado / 1.573 m alt. / En: Maleza Oct-1991 / G. Abril R.”; two reticulate paratypes: the same data; spotted paratype “Colombia, Antioquia, / La Estrella 1.764 m alt. / En: *Piperus* sp. / Abr-1985 V.A. Cortés M.”; spotted paratype: “Colombia, Caldas, / Versalles / En: *Pinnus patula* / Jun-1991 G. Abril R.” (holotype and three paratypes preserved in the Museo Entomológico UNAB, Universidad Nacional de Colombia, Bogotá, Colombia, one paratype in the Department of Biodiversity and Evolutionary Taxonomy, Zoological Institute, University of Wrocław, Wrocław, Poland).

#### Diagnosis.

Black antennae, rounded basal pronotal angles, large scutellum, indistinct sexual dimorphism, venter of pronotum without antennal grooves, short last segment of tarsi, short prosternal collar, antennae with five basal glabrous segments and antennomeres 4 and 5 approximately as long as antennomere 3 place this species in the genus *Cyrtonota*. *Cyrtonota abrili* belongs to the group of species with dorsum without metallic tint. The group was keyed by Borowiec (2007a, with a modification in 2009a) and characters of *Cyrtonota abrili* lead to the couplet 13:

**Table:** 

13	Pronotum with two spots of extremely dense vestiture	**14**
–	Pronotum without spots of extremely dense vestiture	**16**
14	Suture and humeral calli partly or completely black	15
–	Suture and humeral calli the same colour as rest of elytra, fulvous to brown. Peru	***Cyrtonota caprishensis* Sekerka**
15	Pronotum almost semicircular. Ground colour of elytra paler, yellowish-brown. Suture completely black. Basal antennal segments yellowish ventrally. Colombia	***Cyrtonota lurida* (Spaeth)**
–	Pronotum subtrapezoidal. Ground colour of elytra darker, brown. Suture only in anterior part black. Antennae uniformly black. Ecuador	***Cyrtonota aurovestita* (Spaeth)**
16	Elytra with red reticulation or with red reticulate spots	**17**
–	Elytra without red reticulation or red reticulate spots	**20**
17	Elytra on whole surface with red reticulation	18
–	Elytra with red irregular spots or only partly with red reticulation	**17a**
17a	Elytra, on both, disc and explanate margin with irregular reticulation or only explanate margin with large, red, irregular spot. Colombia	**17b**
–	Elytra with six red reticulate spots: two in postscutellar impression, one in the middle of margin of disc and two on slope. Brazil: Bahia	***Cyrtonota bondari* (Spaeth)**
17b	Pronotum more transverse, length/width ratio 2.34. Body stouter, approximately as long as wide, elytral disc forms almost regular, very high gibbosity, punctation of disc very coarse, punctures mostly touching each other, red spot on explanate margin almost round. Colombia	***Cyrtonota pyramidata* (Boheman)**
–	Pronotum less transverse, length/width ratio 1.85–1.97. Body slimmer, longer than wide, elytral disc with high gibbosity in postscutellar area but less convex after the gibbosity, punctation of disc fine, distance between punctures mostly three to four times wider than puncture diameter, red spot on explanate margin elongate. Colombia	***Cyrtonota abrili* sp. n.**

#### Description.

**Figures 1–4.**
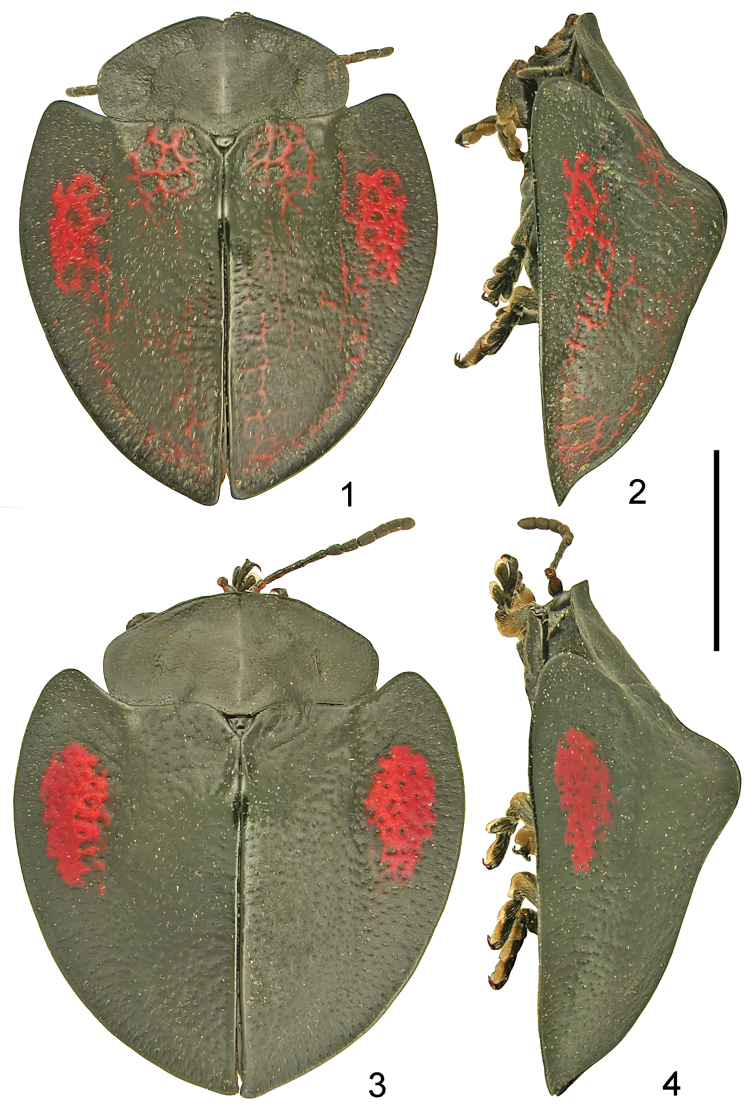
*Cyrtonota abrili* sp. n. **1** holotype dorsal **2** holotype lateral **3** paratype dorsal **4** paratype lateral. Scale bar: 5 mm.

Holotype: length 12.4 mm, width 10.6, length of pronotum 3.3 mm, width of pronotum 6.4 mm, length/width ratio 1.17, width/length ratio of pronotum 1.94; paratypes: length 11.3–13.7 mm, width 10.4–13.2 mm, length of pronotum 3.4–3.7 mm, width of pronotum 6.3–7.2 mm, length/width ratio 1.04–1.14, width/length ratio of pronotum 1.85–1.97. Body stout, elytra regularly rounded on sides, apex of elytra angulate but not elongate or acuminate (Figs 1, 3).

Pronotum and scutellum black. Ground colour of elytra black, in holotype and two paratypes disc with red reticulation in postscutellar impressions, along posterior half of suture and along posterior half of lateral margin of disc, and explanate margin of elytra in anterior third with large red, reticulate spot (Fig. 1); in two other paratypes elytral disc completely black and explanate margin of elytra in anterior third with large red spot without reticulation but with irregular borders and few dark punctures (Fig. 2). Head, antennae, legs and ventrites uniformly black, sometimes last sternite on sides with small, transverse, yellowish-brown spot.

Pronotum transverse, with maximum width approximately in the middle, sides broadly rounded, anterior margin straight or with small triangular emargination in the middle. Surface of disc dull with thin, partly shiny, median, longitudinal line and fine, shallow and very sparse punctation, distance between punctures many times wider than puncture diameter. Explanate margin of pronotum distinctly bordered from disc, on sides with short but deep impression, surface dull, similarly punctate as disc, in some specimens with fine irregular wrinkles.

Scutellum small, triangular, without transverse groove. Base of elytra much wider than pronotum, humeri moderately protruding anterad, humeral angles rounded. Disc very convex, with large but obtuse postscutellar tubercle (Figs 2, 4). Surface in reticulate specimens with thin red reticulation in postscutellar impressions partly extending to latero-basal parts of elytral tubercle, thin red reticulation along both sides of suture and along posterior part of sides of disc, the lateral reticulation joined with red reticulate spot on explanate margin. In dark specimens elytral disc without red reticulation. Dark surface of disc in dark specimens regular, in reticulate specimens slightly irregular with fine and very sparse punctation, dull, distance between punctures mostly three to four times wider than puncture diameter. Whole surface of disc covered with sparse erect setae. Explanate margin of elytra in widest part as wide as half width of disc, in reticulate specimens in anterior third with large, oval, red reticulate spot. In dark specimens the spot is mostly solid, only with irregular margin and several dark punctures but not appears distinctly reticulate. Dark surface of explanate margin regular, dull, punctate similarly as disc. Apex of elytral epipleura without erect hairs.

Ventrites typical for the genus *Cyrtonota*, without diagnostic characters. Genitalia not dissected, in the genus *Cyrtonota* male genitalia are not diagnostic, and spermathecae are not diagnostic within genera of the tribe Mesomphaliini (Borowiec and Opalińska 2007 and unpublished data).

#### Etymology.

Named after the collector, G. Abril, of four of the five specimens of the type series.

#### Distribution.

Antioquia and Caldas departments of north-western Colombia.

#### Ecology.

Little known. The holotype was collected from undergrowth, one paratype on *Piper* sp. and another paratype on *Pinus patula* Schiede ex Schltdl. & Cham. but these plants are unlikely to be the true hosts because the genus *Cyrtonota* is associated with Convolvulaceae plants as far as is known (Borowiec and Świętojańska 2014).

#### Remarks.

*Cyrtonota abrili* is easy to identify using the key presented above. Specimens with red reticulation can be misidentified with small specimens of *Cyrtonota pavens* (Spaeth), and maculate specimens of the new species at first glance are similar to *Cyrtonota deliciosa* (Baly) but both relatives belong to the group of species with elongate or acuminate apex of elytra (couplets 2–12 in Borowiec’s (2007a) key) and both are distinctly larger with length 14–19 mm; *Cyrtonota pavens* differs also in elytra without erect setae. We treated both reticulate and maculate forms as variablity of a single species because similar polymorphism is observed in other reticulate Andean *Cyrtonota* and *Stolas* species i.e. *Cyrtonota bergeali* Borowiec & Sassi, *Stolas cruentata* (Erichson) or *Stolas pellicula* (Spaeth). Other characters such as general body shape, size, punctation, sculpture of surface, vestiture, shape of pronotum, and elytral convexity are the same in both forms.

### New faunistic data

***Acromis sparsa* (Boheman, 1854)**

**Antioquia**, Carepa, Granja Tulenapa, 28 m, 7°46'00"N/76°39'58"W, 13 IX 2001, 1 ex., R. Delgado; **Cundinamarca**, Guaduas, 1007 m, 5°04'12"N/74°35'52"W, 14 X 1995, 1 ex., S. Sánchez; **Cundinamarca**, Vega, 1215 m, 4°59'57"N/74°20'23"W, 8 VII 1976, 1 ex., F. Garcia; **Norte de Santander**, Cúcuta, 320 m, 7°53'N/72°30'W, 13 VI 1975, 1 ex., J. Yañez; **Santander**, La Belleza, Vrda. Los Naranjos, 1900 m, 5°51'57"N/73°58'02"W, 21 III 1997, 1 ex., H. Marin; **Tolima**, Flandes, 290 m, 4°17'N/74°49"W, 27 III 1975, 1 ex., A. Mendoza; **Tolima**, Mariquita, 328 m, 5°12'N/74°55'W, 9 VI 1972, 1 ex., M. Villamizar, 10 VI 1972, 2 ex., F. Mora.

**Distribution.** Bolivia; Brazil; Colombia; Costa Rica; southern Mexico; Nicaragua; Panama; Peru; Venezuela.

***Agroiconota judaica* (Fabricius, 1781)**

**Antioquia**, Cocorná, 1288 m, 6°03'25"N 75°11'07"W, VI 1977, 1 ex., R. Vélez; **Antioquia**, Concepción, 1862 m, 6°23'55"N/75°15'22"W, II 1997, 1 ex., F.J. Serna & J.G. Hurtado; **Antioquia**, Fredonia, 1317 m, 5°55'28"N/75°40'51"W, VIII 1985, 1 ex., P.E. Mejia; **Antioquia**, Medellin, 1486 m, 6°13'N/75°34'W, IX 1963, 1 ex., F. Gallego; **Antioquia**, Puerto Triunfo, Rio Claro, 115 m, 5°52'N/74°38'W, V 1985, 1 ex., A. Madrigal; **Antioquia**, Sabaneta, 1609 m, 6°09'11"N, 75°17'19"W, 17 VII 1968, 1 ex., G. Bustamante; **Boyacá**, Turmequé, 2404 m, 5°19'48"N/73°29'42"W, 10 IX 1995, 1 ex., T. Corredor; **Córdoba**, Ayapel, 22 m, 8°19'N, 75°09'W, 1 IV 1969, 1 ex., 4 IV 1969, 1 ex., 12 VI 1969, 1 ex., R. Abisambra; **Cundinamarca**, Agua de Dios, San Marcos, 600 m, 4°22'N/74°40'W, 10 III 2003, 1 ex., L. Moreno & F. Escobar; **Cundinamarca**, Caqueza, Girón de Blanco, 1740 m, 4°24'N/73°53'W, 15 IX 2003, 1 ex., F. Rodriguez; **Cundinamarca**, Fomeque, 1895 m, 4°29'22"N/73°53'54"W, 4 III 2005, 1 ex., N. Rojas; **Cundinamarca**, Fusagasugá, 1746 m, 4°20'49"N/74°21'53"W, I 1980, 1 ex., C. Rojas; **Cundinamarca**, Guayabetal, 1200 m, 4°13'N/73°48'W, 1 V 1969, 1 ex., L.M. Rico & C. Cujia, VI 1969, 1 ex., A. Uribe; **Cundinamarca**, Guayabetal, Vrda. Susumaco, ribera del Rio Negro, 1200 m, 4°13'40"N/73°48'59"W, 11 IV 1993, 1 ex., A. Diego; **Cundinamarca**, Guayabetal de Siquima, 1630 m, 4°52'N/74°28'W, 1 V 1969, 1 ex., A. Silva; **Cundinamarca**, La Mesa, 1298 m, 4°38'05"N, 74°27'57"W, 27 X 1996, 1 ex., S. Olarte; **Cundinamarca**, La Mesa, Laguna Pedro Palo, 1298 m, 4°38'05"N, 74°27'57"W, 13 IX 1997, 1 ex., C. Bejarano, J. Diaz & E. Guzmán; **Cundinamarca**, La Palma, 1462 m, 5°21'51"N/74°23'51"W, 1 ex., A. Pinilla; **Cundinamarca**, Quétame, 1531 m, 4°19'N/73°50'W, VIII 1961, F. Gallego; **Cundinamarca**, Sasaima, 1225 m, 4°57'N/76°26'W, 20 X 1971, 1 ex., A. Coronel; **Cundinamarca**, Usme, 2960 m, 4°28'24"N/74°07'44"W, 8 X 1995, 1 ex., T. Corredor; **Cundinamarca**, Villeta, 842 m, 5°00'52"N/74°28'23"W, 16 III 1968, 1 ex., J. Quintero, 10 XI 2003, 1 ex. G. Herrera; **Cundinamarca**, Viotá, 567 m, 4°26'31"N/74°31'33"W, 20 VII 1976, 5 exx., J. López; **Cundinamarca**, Viotá, El Triunfo, 567 m, 4°26'N/74°31'W, 11 IV 1997, 1 ex., M. Mora; **Meta**, Cubarral, San Luis de Cubarral, Brisas del Ariari, 180 m, 3°47'N/73°52'W, 6 IV 2004, 1 ex., L. Ramirez & A. Vargas; **Meta**, La Macarena, 3°11'16"N/734°59'20"W, 10 IX 1996, 1 ex., O. Trujillo; **Meta**, Lejanias, 1000 m, 3°30'49"N/74°03'06"W, 15 V 1994, 1 ex., Peña & Martinez; **Meta**, Puerto Gaitán, 149 m, 4°19'04"N/72°03'17"W, 15 IX 2001, 1 ex., M. Rozo, 26 V 2005, 1 ex., J. Castro; **Meta**, Villavicencio, 467 m, 4°09'N/73°39'W, 10 V 1972, 1 ex., E. Daza, 22 VI 1974, 1 ex., E. Torres, 1 X 1975, 1 ex., O. Rodriguez, 15 VI 1979, 1 ex., W. Guevara, 8 XI 2002, 1 ex., J. Rodriguez, 500 m, 6 X 2003, 1 ex., J. Rodriguez; **Meta**, Villavicencio, Rio Tigre, 467 m, 4°09'N/73°39'W, 21 XI 2003, 1 ex., A. Molano; **Tolima**, Honda, 229 m, 5°12'25"N/74°44'28"W, 15 VI 1971, 1 ex., L. Diaz; **Tolima**, Purificación, 310 m, 3°51'N/74°56'W, 9 IV 1972, 1 ex., D. Bonilla; **Valle del Cauca**, Caicedonia, 1000 m, 4°19'N, 75°50'W, 17 X 2001, 1 ex., C.M. Ospina; **Valle del Cauca**, Miranda, 1111 m, 3°15'19"N/76°13'54"W, 20 VI 1976, 1 ex., H. Durán.

**Distribution.** Bolivia; Brazil; Colombia; Costa Rica; Ecuador; French Guyana; Guyana; Nicaragua; Panama; Paraguay; Peru; Surinam; Trinidad and Tobago; Venezuela.

***Agroiconota propinqua* (Boheman, 1855)**

**Antioquia**, Rionegro, 2137 m, 6°09'20"N, 75°22'58"W, 4 IV 1977, 1 ex., L. Zapata; **Caldas**, La Dorada, 178 m, 5°27'N/74°40'W, 11 XII 1965, 1 ex., Pieschacon; **Casanare**, Yopal, 350 m, 5°21'N/72°24'W, 12 X 2003, 1 ex., N. Pachón & R. Ospinañ; **Chocó**, Titumate, 12 m, 8°18'N/77°04'W, IV 1980, 1 ex., E. Urueta; **Cundinamarca**, Agua de Dios, 552 m, 4°22'04"N/74°40'26"W, 2 X 1998, 1 ex., S. Serna; **Cundinamarca**, Anapoima, 698 m, 4°33'18"N/74°32'13"W, 20 XI 1993, 1 ex., P. Numpaque; **Cundinamarca**, Apulo, 421 m, 4°31'18"N/74°35'58"W, 7 IV 1968, 1 ex., F. Santacruz, 4 III 1981, 1 ex., M. Ortega & G. Cavalier; **Cundinamarca**, Bogotá, 2620 m, 4°35'56"N/74°04'51"W, 14 III 1981, 1 ex., 19 XI 1981, 1 ex., Vargas; **Cundinamarca**, Cachipay, Insp. Pol. Anolaima, 5°16'22"N/74°34'22"W, 13 III 1991, 1 ex., G. Castiblanco; **Cundinamarca**, Fusagasugá, 1746 m, 4°20'N/74°21'W, 8 V 1975, 1 ex., R. Puentes; **Cundinamarca**, Girardot, 281 m, 4°18'18"N/74°48'08"W, 12 X 1992, 1 ex., A. Escobar; **Cundinamarca**, Guayabetal, 1200 m, 4°13'40"N/73°48'59"W, 15 III 1969, 1 ex., P. Corzo, 24 VI 1973, 1 ex., R. Madero, 13 V 1974, 1 ex., C. Sierra; **Cundinamarca**, La Mesa, 1298 m, 4°38'05"N, 74°27'57"W, 19 IX 2003, 1 ex., M. Pinzón; **Cundinamarca**, La Mesa, Laguna Pedro Palo, 1298 m, 4°38'05"N, 75°11'7"W, 21 XI 1997, 1 ex., M. Camargo; **Cundinamarca**, La Vega, 1215 m, 4°59'N/74°20'W, 15 V 1996, 1 ex., J. Ardila; **Cundinamarca**, Tena, 1384 m, 4°39'33"N/74°23'28"W, 18 VIII 1998, 1 ex., S. Serma; **Cundinamarca**, Tocaima, 400 m, 4°27'40"N/74°38'10"W, 8 V 1972, 1 ex., Valero & Saldaña, 23 III 1994, 1 ex., N. Pinzón; **Cundinamarca**, Villeta, 842 m, 5°00'52"N/74°28'23"W, 23 V 1968, 1 ex., A. Duarte; **Magdalena**, Pivijai, 7 m, 10°27'N/74°36'W, VI 1985, 3 exx., A. Madrigal; **Meta**, Villavicencio, 467 m, 4°09'N/73°39'W, 20 VI 1967, 1 ex., E. Peralta; **Tolima**, Ambalema, 285 m, 4°47'N/74°46'W, 17 III 1983, 1 ex., Franco & Rodriguez; **Tolima**, Guamo, 323 m, 4°02'05"N/74°58'25"W, 10 V 1994, 1 ex., M. Peñafort; **Tolima**, Ibagué, 1285 m, 4°26'N/75°14'W, 6 V 1998, 1 ex., J. Jaramillo, 10 IV 1999, 1 ex., Trisdt; **Tolima**, Prado, 321 m, 3°45'11"N/74°55'59"W, 26 XI 1989, 1 ex., M. Beltrán; **Tolima**, Saldaña, 310 m, 3°56'05"N/75°01'13"W, 2 IV 1997, 1 ex., A. Ariza & L. Ferrucho.

**Distribution.** Colombia; Costa Rica; Cuba; Dominican Republic; Haiti; Jamaica; Nicaragua; Panama; Puerto Rico; Venezuela.

***Canistra osculatii* Guérin, 1855**

**Risaralda**, Pereira, 2137 m, 4°49'02"N/75°41'54"W, 27 XI 1996, 1 ex., R. Humberto.

**Distribution.** Brazil; Colombia; Ecuador; Peru.

***Charidotella balteata* (Champion, 1894)**

**Antioquia**, San Antonio de Prado, 1955 m, 6°11'N/75°39'W, IX 1981, 2 exx., G. Morales.

**Distribution.** Panama. **New to Colombia**.

***Charidotella carnulenta* (Erichson, 1847)**

**Caqueta**, San Vicente del Caguan, Vda. El Tigre, 480 m, 2°07'N/74°46'W, 25 III 2002, 1 ex., J. Méndez & E. Garcia; **Meta**, Villavicencio, 467 m, 4°09'N/73°39'W, 24 V 1969, 1 ex., J.R. Alba.

**Distribution.** Argentina; Bolivia; Colombia; Peru; Venezuela.

***Charidotella circumnotata* (Boheman, 1862)**

**Antioquia**, San Luis, 1050 m, 6°02'N/74°59'W, I 1986, VII 1983, 1 ex., G. Morale; **Huila**, Neiva, 442 m, 2°55'46"N/75°17'31"W, 13 IV 1967, 1 ex., H. Ramos; **Santander**, Bucaramanga, 958 m, 7°07'17"N/73°07'33"W, 11 IV 1990, 1 ex., C. Sarmiento.

**Distribution.** Bolivia; Brazil; Costa Rica; Ecuador; French Guyana; Nicaragua; Panama; Peru. **New to Colombia**.

***Charidotella glaucovittata* (Erichson, 1847)**

**Cundinamarca**, Guayabetal, 1200 m, 4°13'N/73°48'W, 1 V 1969, 1 ex., L.M. Rico & C. Cujia; **Meta**, Puerto López, 181 m, 4°05'N/72°58'W, 4 X 1974, 1 ex., J. Britton; **Valle del Cauca**, Jamundi, 975 m, 3°15'N/76°32'W, 23 VI 1972, 1 ex., E. Leaño.

**Distribution.** Bolivia; Ecuador; Paraguay; Peru. **New to Colombia**.

***Charidotella immaculata* (Olivier, 1790)**

**Cundinamarca**, Sasaima, 1225 m, 4°57'N/76°26'W, 5 III 1971, 1 ex., L. Morales; **Cundinamarca**, Tibacuy, Ins. Pol Cumaca, 1647 m, 4°21'N/74°27'W, 18 XI 1994, 1 ex., Reina; **Cundinamarca**, Villeta, 804 m, 5°00'52"N/74°28'23"W, 17 VI 1966, 1 ex., E. Aponte; **Huila**, Timaná, 1010 m, 1°58'N/75°56'W, 25 VI 1971, 1 ex., L. Morales; **Meta**, Cubarral, San Luis de Cubarral, Brisas del Ariari, 180 m, 3°47'N/73°52'W, 9 IV 2004, 1 ex., L. Ramirez & A. Vargas; **Tolima**, Melgar, 323 m, 4°12'24"N/74°38'44"W, 19 III 1969, 1 ex, C. Forero.

**Distribution.** Argentina; Bolivia; Brazil; Dominica; Colombia; Ecuador; French Guyana; Paraguay; Peru; Surinam; Trinidad and Tobago; Venezuela.

***Charidotella incorrupta* (Boheman, 1855)**

**Meta**, Villavicencio, 467 m, 4°09'N/73°39'W, 1 X 1975, 1 ex., C. Rodriguez; **Tolima**, Espinal, 322 m, 4°09'N/74°53'W, 27 IV 1969, 1 ex., E. Orjuela.

**Distribution.** Bolivia; Brazil; Colombia; Costa Rica; Ecuador; Panama; Peru; Venezuela.

***Charidotella liquida* (Erichson, 1847)**

**Cundinamarca**, Sasaima, 1225 m, 4°57'N/76°26'W, 8 III 1975, 1 ex., A. Alarcón; **Meta**, Cubarral, Finca Brisas del Ariari, 180 m, 3°47'N/73°52'W, 21 XI 2003, 1 ex., L. Pérez.

**Distribution.** Bolivia; Peru. **New to Colombia**.

***Charidotella moraguesi* Borowiec, 2007b**

**Tolima**, Carmen de Apicalá, Finca La Ponderosa, 328 m, 4°09'00"N/75°46'37"W, 23 X 1998, 1 ex., H. Parada.

**Distribution.** French Guyana. **New to Colombia**.

***Charidotella puella* (Boheman, 1855)**

**Antioquia**, Carepa, Granja Tulenapa, 28 m, 7°46'00"N/76°39'58"W, 14 IX 2001, 1 ex., G. Morales; **Antioquia**, Puerto Triunfo, Rio Claro, 115 m, 5°52'N/74°38'W, V 1985, 1 ex., A. Madrigal, VI 1985, 1 ex., R. Vélez; **Meta**, Acacias, 522 m, 4°00'N/73°46'W, 19 VIII 1976, 1 ex., S. Rodriguez; **Cundinamarca**, Tocaima, 400 m, 4°27'N/74°38'W, 2 XI 1973, 1 ex., O. Barbosa; **Meta**, Villavicencio, 467 m, 4°09'N/73°39'W, 1 X 1975, 1 ex., C. Rodriguez; **Tolima**, Armero, 352 m, 4°57'N/74°54'W, 14 IX 1980, 1 ex., E. Maldonado.

**Distribution.** Belize; Colombia; Costa Rica; Ecuador; French Guyana; Mexico; Honduras; Nicaragua; Panama; Peru; Venezuela.

***Charidotella sexpunctata* (Fabricius, 1781)**

**Antioquia**, Concepción, 1862 m, 6°23'55"N/75°15'22"W, II 1997, 1 ex., F.J. Serna & J.G. Hurtado; **Antioquia**, San Luis, 1050 m, 6°02'50"N/74°59'50"W, IV 1995, 1 ex., Silva; **Cundinamarca**, Apulo, 421 m, 4°31'N/74°35'W, 8 VI 1984, 1 ex., Morales; **Cundinamarca**, Bogotá, 2620 m, 4°35'56"N/74°04'51"W, 30 V 1990, 1 ex., B. Diaz; **Cundinamarca**, Fusagasugá, 1746 m, 4°20'N/74°21'W, 4 III 1990, 1 ex., A. Pagos; **Cundinamarca**, Girardot, 150 m, 4°30'N/75°45'W, 7 XI 1970, 1 ex., A. Suárez, 24 XI 2001, 1 ex., D. Moreno; **Cundinamarca**, Guaduas, 1007 m, 5°04'N/74°35'W, 30 VI 1966, 1 ex., M. Pelaez, no date, 1 ex., O. Moro; **Cundinamarca**, Guayabetal, 4°13'40"N/73°48'59"W, 14 X 1970, 1 ex., H. Hernández; **Cundinamarca**, La Mesa, 1298 m, 4°38'05"N/74°27'57"W, 10 IX 1994, 1 ex., A. Pinilla; **Cundinamarca**, La Mesa, San Javier, 1298 m, 4°38'05"N/74°27'57"W, 1 VI 1997, 1 ex., A. Alessandri; **Cundinamarca**, La Mesa, Vda. San Joaquin, 670 m, 4°38'05"N/74°27'57"W, 17 IV 2002, 1 ex., J. Gómez; **Cundinamarca**, La Palma, 1462 m, 5°21'51"N/74°23'51"W, 28 IX 1973, 1 ex., A. Pinilla; **Cundinamarca**, Manta, 1924 m, 5°00'42"N/73°32'41"W, 3 V 1997, 1 ex., X. Medina; **Cundinamarca**, Silvania, 1470 m, 4°24'21"N/74°23'24"W, 2 XI 1994, 2 exx., C. Ferro, 15 III 1997, 1 ex., C. Bojacá; **Cundinamarca**, Villeta, Vda. Topacio, 842 m, 5°00'N/74°28'W, 31 X 1970, 1 ex., L. Sánchez; **Cundinamarca**, Viotá, 567 m, 4°26'31"N/74°31'33"W, 6 VI 1989, 1 ex., F. Ramirez; **Huila**, Neiva, 442 m, 2°55'46"N/75°17'31"W, 25 V 1974, 1 ex., C. Reyes; **Meta**, Acacias, 522 m, 4°00'N/73°46'W, 1 V 1974, 1 ex., I. Oviedo, 28 III 1975, 1 ex., B. Correa; **Meta**, Guamal, 518 m, 3°51'N/73°45'W, 2 VIII 1968, 1 ex., S. Bobadillo, 21 V 1994, 1 ex., López & Rico; **Meta**, Puerto López, 450 m, 4°06'01"N/72°57'22"W, 27 V 2005, 1 ex., J. Castro, 26 V 2005, 1 ex., D. Rios; **Meta**, Villavicencio, 467 m, 4°09'N/73°39'W, 8 VI 1969, 1 ex., S. Martinez, 28 VI 1970, 1 ex., H. Osorio; **Santander**, Bucaramanga, 958 m, 7°07'17"N/73°07'33"W, 2 XI 1973, 1 ex., Castellano; **Santander**, La Belleza, 1900 m, 5°51'N/73°58'W, 28 III 1972, 1 ex., L. Angulo; **Santander**, Socorro, 1219 m, 6°25'N/73°14'W, 1 ex., L. Arenas & R. Lesmes; **Tolima**, El Guamo, 323 m, 4°02'05"N/74°58'25"W, 16 X 2001, 1 ex., A. Sarmiento; **Tolima**, Espinal, 322 m, 4°09'N/74°53'W, 19 V 2003, 1 ex., M. Capera & C. Jerez; **Tolima**, Honda, 229 m, 5°12'N/74°44'W, 3 IV 1974, 1 ex., E. Guevara; **Tolima**, Ibagué, 1285 m, 4°26'50"N/75°14'44"W, 30 I 1981, 1 ex., L. Lueñas; **Tolima**, Melgar, 323 m, 4°12'24"N/74°38'44"W, 1 X 1975, 1 ex, L. Méndez; **Tolima**, Purificación, 310 m, 3°51'N/74°56'W, 4 IV 1969, 1 ex., B. Vásquez; **Valle del Cauca**, Caicedonia, 1400 m, 4°19'25"N, 75°50'00"W, 16 X 2001, 1 ex., C. Prada.

**Distribution.** widespread from Canada to northern Argentina, include Brazil.

***Charidotella tuberculata* (Fabricius, 1775)**

**Meta**, Granada, 450 m, 3°32'N/73°43'W, 20 IX 1989, 1 ex., G. Villalba; **Tolima**, Falán, 998 m, 5°07'35"N/74°57'18"W, 10 IV 1990, 1 ex., Y. Beltrán.

**Distribution.** Colombia; Costa Rica; El Salvador; Guatemala; Honduras; Mexico; Nicaragua; Venezuela.

***Charidotella vinula* (Boheman, 1855)**

**Cundinamarca**, Sasaima, 1225 m, 4°57'59"N/76°26'15"W, 23 V 1967, 1 ex., E. Delgado; **Cundinamarca**, Viotá, El Triunfo, 567 m, 4°26'N/74°31'W, 10 III 1968, 1 ex., S. Sánchez; **Valle del Cauca**, La Unión, Fca. Grajales, 1250 m, 4°35'N 76°15'W, 14 X 2003, 1 ex., A. Molano.

**Distribution.** Argentina; Bolivia; Brazil; Colombia; Ecuador; French Guyana; Guyana; Mexico; Paraguay; Surinam; Venezuela.

***Charidotis bipartita* (Boheman, 1855)**

**Santander**, Puerto Wilches, 75 m, 7°20'52"N/73°54'25"W, 15 I 1993, 1 ex., G. Vargas.

**Distribution.** Brazil; French Guyana; Honduras; Panama; Venezuela. **New to Colombia**.

***Charidotis cincticula* (Boheman, 1855)**

**Cundinamarca**, Villeta, 842 m, 5°00'52"N/74°28'23"W, 28 V 1990, 1 ex., R. Suárez.

**Distribution.** Bolivia; Brazil; Ecuador; French Guyana; Peru. **New to Colombia**.

***Charidotis vitreata* (Perty, 1830)**

**Antioquia**, Amalfi, Cañón del Porce, Calandria, 985 m, 6°55'N/75°04'W, 14 I 1998, 1 ex., J. Hurtado.

**Distribution.** Argentina; Brazil; Colombia; Guatemala; Mexico; Nicaragua; Panama; Peru.

***Chelymorpha cavata* Boheman, 1854**

**Cundinamarca**, Guayabetal, 1200 m, 4°13'N/73°48'W, 1 V 1969, 1 ex., L.M. Rico & C. Cujia.

**Distribution.** Colombia; Venezuela.

***Chelymorpha marginata* (Linnaeus, 1758)**

**Cesar**, Valledupar, 182 m, 9°29'N/73°15'W, 5 IV 1972, 1 ex., J. Avellano; **Cundinamarca**, Guayabetal, 1200 m, 4°13'N/73°48'W, 1 V 1969, 1 ex., C. Cujia, 11 V 1972, 1 ex., E. Daza; **Cundinamarca**, Medina, 431 m, 4°38'54"N/73°19'37"W, 14 X 1989, 1 ex., C. Franco; **Cundinamarca**, Tocaima, 400 m, 4°27'N/74°38'W, 17 V 1969, 1 ex., A. Guzmán.

**Distribution.** Bolivia; Brazil; Colombia; Ecuador; French Guyana; Paraguay; Surinam; Venezuela.

***Chelymorpha testaceomarginata* Boheman, 1854**

**Boyacá**, Sogamoso, 2569 m, 5°42'58"N/72°55'38"W, 8 VI 1967, 1 ex., Plazas; **Cundinamarca**, Bogotá, 2620 m, 4°35'56"N/74°04'51"W, 3 IV 1969, 1 ex., Plazas; **Cundinamarca**, Girardot, 281 m, 4°18'18"N/74°48'08"W, 6 XI 1989, 1 ex., E. Gonzáles; **Cundinamarca**, Guaduas, 1007 m, 5°04'N/74°35'W, 27 IX 1975, 1 ex., D. Moreno; **Cundinamarca**, La Mesa, San Javier, 1298 m, 4°38'05"N/74°27'57"W, 1 ex., Barbosa & Garcés,1110 m, 4°38'N/74°27'W, 2 IV 1972, 1 ex., G. Arguelles; **Cundinamarca**, Pacho, 1798 m, 5°07'57N/74°09'42"W, 20 V 1992, 1 ex., R. Chizaba, 8 IV 1995, 1 ex., P. Carlos; **Cundinamarca**, Villeta, 842 m, 5°00'N/74°28'W, 12 XI 1981, 1 ex., C. Orjuela; **Huila**, Neiva, 442 m, 2°55'N/75°17'W, 10 VII 1972, 1 ex., F. Gutierez; **Meta**, Villavicencio, 467 m, 4°09'N/73°39'W, 6 X 1975, 1 ex., J. Gómez; **Norte de Santander**, Cúcuta, 320 m, 7°53'N/72°30'W, 14 IX 1974, 1 ex., L. Ojeda; **Tolima**, Mariquita, 328 m, 5°12'N/74°55'W, 22 VI 1976, 1 ex., J. Nieto; **Valle del Cauca**, Buga, 969 m, 3°54'06"N/76°18'14"W, 20 IV 1994, 1 ex., Lizarrazo & Barriga.

**Distribution.** Colombia; Costa Rica; Dominican Rep.; Panama; Venezuela, N Brazil. Probably many records of *Chelymorpha cribraria* (F.) from northwestern part of South America belongs to *Chelymorpha testaceomarginata* Boh.

***Chersinellina heteropunctata* (Boheman, 1854)**

**Córdoba**, Monteria, Tres Palmas, 18 m, 8°29'N/75°56'W, VI 1973, 1 ex., R. Vélez; **Cundinamarca**, Fusagasugá, 1746 m, 4°20'49"N/74°21'53"W, X 1994, 1 ex., M. Ahumada.

**Distribution.** Colombia; Panama.

***Cistudinella foveolata* Champion, 1894**

**Guaviare**, San Jose del Guaviaré, 240 m, 2°33'N/72°38'W, III 1996, 1 ex., C. Forero.

**Distribution.** Colombia; Costa Rica; Ecuador; Panama.

***Coptocycla* n. sp. Sekerka & Windsor, in prep.**

*Coptocycla dorsoplagiata* Champion, 1894 (ex parte).

*Coptocycla rufonotata* sensu Spaeth, 1936c: 255; Windsor et al. 1992: 390; Borowiec 1996: 225 (as *Psalidonota rufonotata*) all misinterpretations.

**Antioquia**, Jericó, 1967 m, 5°47'39"N/75°47'23"W, 1996, 1 ex., C. Tamayo.

**Distribution.** Colombia; Costa Rica; Panama; Venezuela.

Note: Spaeth (1936c) misinterpreted *Coptocycla rufonotata* Champion, 1893 and recent records of this taxon (Windsor et al. 1992: 390; Borowiec 1996: 225) concern an undescribed species. Its formal description is now under preparation by Sekerka and Windsor in their review of the Panamanian tortoise beetles.

***Cteisella divalis* Spaeth, 1926b**

**Cundinamarca**, Girardot, 150 m, 4°30'N/75°45'W, 19 III 1969, 1 ex., Junca; **Tolima**, Honda, 229 m, 5°12'25"N/74°44'28"W, 8 V 1966, 1 ex., E. Guzmán, 1 ex., O. Pedraza.

**Distribution.** Colombia; Panama; Venezuela.

***Cyrtonota bergeali* Borowiec & Sassi, 1999**

**Valle del Cauca**, Calima, C. Alegre, 1414 m, 3°55'N, 76°40'W, IV 1990, 2 exx., L.C. Pardo-Locamo.

**Distribution.** known only from Valle del Cauca in Colombia.

***Cyrtonota dissecta* (Boheman, 1850)**

**Cundinamarca**, San Antonio del Tequendama, 1521 m, 4°37'04"N/74°21'15"W, 27 IV 1990, 1 ex., M. Fuentes; **Cundinamarca**, Viotá, 567 m, 4°26'31"N/74°31'33"W, 10 V 1966, 1 ex., P. Mendoza; **Risaralda**, Ucumari, vereda El Bosque, V 2000, 1 ex., J. Sáenz.

**Distribution.** Colombia; Peru.

***Cyrtonota goryi* (Boheman, 1850)**

**Meta**, Villavicencio, 467 m, 4°09'N/73°39"W, 24 V 1969, 1 ex., J.R. Alba.

**Distribution.** known only from Colombia.

***Cyrtonota moderata* (Spaeth, 1913)**

**Caldas**, Guática, 1820 m, IX 1994, 1 ex., A. Madrigal; Quindio, Armenia, 1483 m, 4°31'N/75°42"W, I 2000, 1 ex., A. Madrigal.

**Distribution.** known only from Colombia.

***Cyrtonota steinheili* (Wagener, 1877)**

**Cundinamarca**, La Mesa, 1298 m, 4°38'05"N/74°27'57"W, 5 IV 1994, 1 ex., H. Rodiguez.

**Distribution.** Colombia; Peru.

***Cyrtonota textilis* (Boheman, 1850)**

**Cundinamarca**, Apulo, 421 m, 4°31'18"N/74°35'58"W, 16 IX 1995, 1 ex., Fredy; **Cundinamarca**, Bojacá, Vda. Bojacá, El Chilcal, 1950 m, 4°44'N/74°20'W, 5 V 2001, A. Osorio & C. Zuluaga; **Cundinamarca**, La Mesa, Laguna Pedro Palo, 1298 m, 4°38'05"N, 75°11'7"W, 20 III 1993, 1 ex., P. Osorio, 2031 m, 4°41'07"N/74°23'50"W, 16 IV 1994, 1 ex., D. Moreno, 1298 m, 2 XI 1996, 1 ex., A. Romero, 2031 m, IX 1998, 1 ex., J. Muñoz; **Cundinamarca**, La Mesa, Laguna Pedro Palo, Quebrada Patio Bonito, 1298 m, 4°38'05"N/74°27'57"W, 1 ex., J. Martinez & D. Vegas; **Cundinamarca**, Pandi, 1024 m, 4°11'40"N/74°29'50"W, 12 X 1995, 1 ex., T. Corredor; **Cundinamarca**, San Antonio del Tequendama, 1500 m, 4°37'N/74°21'W, 1 V 2001, 1 ex., C. Martinez, J. Muñoz & J. Abello; **Cundinamarca**, San Bernardo, 1600 m, 4°11'10"N/74°24'31"W, 19 IX 1989, 1 ex., M. Pava; **Cundinamarca**, Soacha, 2568 m, 4°35'03"N/74°13'23"W, 2 IX 1989, 1 ex., A. Martinez; **Cundinamarca**, Tena, 1384 m, 4°39'33"N/74°23'28"W, 17 XI 1993, 1 ex., H. Rondón, 30 III 1994, 2 ex., A. Tovar; **Cundinamarca**, Villeta, Vda. Topacio, 842 m, 5°00'N/74°28'W, 6 IV 2001, 1 ex., A. Afanador & C. Berdugo; Guainia, Puerto Inirida, 100 m, 3°49'N/67°52'W, 16 V 1992, 1 ex., S. Bernal; **Huila**, Neiva, 442 m, 2°55'46"N/75°17'31"W, 1 ex., H. Trujillo; **Meta**, Granada, 450 m, 3°32'N/73°43'W, 1 V 1994, 1 ex., N. Pinzón; **Meta**, Villavicencio, 467 m, 4°9'N/73°39'W, 3 IV 1994, 1 ex., C. Páez; **Tolima**, Cajamarca, El Tigre, 1814 m, 4°26'N/75°25'W, 27 V 2002, 1 ex., J. Martinez; **Tolima**, Guamo, 323 m, 4°02'05"N/74°58'25"W, 20 V 1995, 1 ex., T. Rene; **Tolima**, Ibagué, 1285 m, 4°26'50"N/75°14'44"W, 18 IX 1998, 1 ex., A. Rubio; **Valle del Cauca**, Sevilla, 1612 m, 4°16'06"N/75°57'13"W, 3 I 1990, 1 ex., M. Caro

**Distribution.** known only from Colombia.

***Delocrania cossyphoides* Guérin, 1844**

**Magdalena**, San Sebastián de Buenavista, 25 m, 9°23'N/74°11'W, 27 II 1994, 4 exx. on Palma ornamental, A. Madrigal; **Santander**, Puerto Wilches, 75 m, 7°20'52"N/73°54'25"W, 2 VIII 2004, 1 ex., D. Ávila.

**Distribution.** Brazil; Trinidad; Venezuela. **New to Colombia**.

***Deloyala fuliginosa* (Olivier, 1790)**

**Córdoba**, Monteria, 19 m, 8°45'N/75°52'W, IX 1972, 1 ex., R. Vélez.

**Distribution.** Belize; Brazil; Colombia; Costa Rica; Cuba; Dominican Republic; El Salvador; Guatemala; Martinique; Mexico; USA: Texas; Nicaragua; Panama; Venezuela.

***Deloyala insubida* (Boheman, 1855)**

**Antioquia**, Amalfi, Cañón del Porce, 1050 m, 6°55'N/75°04'W, 1997, 1 ex., J. Hurtado; **Cundinamarca**, Arbeláez, 1417 m, 4°16'N/74°24'W, 1 XI 1973, 1 ex., A. González; **Cundinamarca**, La Mesa, 1298 m, 4°38'05"N/74°27'57"W, 8 IV 1970, 1 ex., L. Moreno, 1 IV 1997, 1 ex., J. Camargo; **Cundinamarca**, La Mesa, via a Mesitas, 1298 m, 4°38'05"N/74°27'57"W, 19 VI 1998, 1 ex., C. Navas; **Meta**, Villavicencio, Puerto Lopez, 450 m, 3°08'N/73°45'W, 26 V 2005, 1 ex., D. Rios; **Tolima**, Armero, 352 m, 4°57'N/74°54'W, 10 XII 1965, 1 ex., Pérez; **Tolima**, Ibagué, 1285 m, 4°26'50"N/75°14'44"W, 25 IV 1978, 1 ex., G. Vargas; **Tolima**, Melgar, 323 m, 4°12'24"N/74°38'44"W, 31 III 1972, 1 ex, M. Acosta.

**Distribution.** Colombia; Costa Rica; Ecuador; Panama; Venezuela.

***Discomorpha amazona* (Spaeth, 1940)**

**Meta**, Cumaral, 480 m, 4°17'N/73°33'W, 29 IX 1973, 1 ex., A. Vargas; **Meta**, Villavicencio, 469 m, 4°09'N/73°39'W, 14 X 1994, 1 ex., F. Montes; **Santander**, Oiba, Vda. Pedregal, 1452 m, 6°15'N/73°15'W, 4 VI 2003, 1 ex., J. Cárdenas.

**Distribution.** Colombia; Peru.

***Discomorpha batesi* (Boheman, 1856)**

**Antioquia**, Ituango, 1675 m, I 1989, 1 ex., D. Calle.

**Distribution.** Brazil; Colombia; Peru.

***Discomorpha biplagiata* (Guérin-Méneville, 1844)**

**Casanare**, Mani, 200 m, 4°49'N/72°17'W, 22 III 1967, 2 exx., Plazas; **Casanare**, Yopal, 350 m, 5°21'N/72°24'W, 5 V 1967, 1 ex., H. Lizarazo; **Cundinamarca**, Girardot, 281 m, 4°18'18"N/74°48'08"W, 31 VIII 1924, 1 ex., J. Rincón; **Cundinamarca**, Guayabetal, 1200 m, 4°13'N/73°48'W, 4 V 1969, 1 ex., H. Muñoz & O. Millán; **Cundinamarca**, Villeta, 842 m, 5°00'N/74°28'W, 4 VII 1972, 1 ex., A. Canunes; **Huila**, Neiva, 442 m, 2°55'46"N/75°17'31"W, 20 X 1974, 1 ex., J. Ruiz; **Meta**, Cumaral, 480 m, 4°17'N/73°33'W, 5 XII 1988, 1 ex.; **Meta**, Guamal, 518 m, 3°51'N/73°45'W, 19 X 1994, 1 ex., Pinilla; **Meta**, La Macarena, 3°11'16"N/734°59'20"W, 29 III 1997, 1 ex., C. Santana; **Meta**, San Juan de Arama, 450 m, 3°22'N/73°52'W, VIII 1969, 1 ex.; **Meta**, San Martin, 419 m, 3°42'N/73°42'W, 20 VIII 1974, 1 ex., F. Garzón, 30 III 1994, 1 ex., A. Avella; **Meta**, Villavicencio, 467 m, 4°09'N/73°39'W, VI 1967, 2 exx., Cardona, 17 VI 1967, 1 ex., J. Carrillo, 10 V 1969, 1 ex., J. López, 20 VI 1969, 1 ex., M. Barreto, 1 III 1972, 2 exx., I. Garzón, 1 III 1972, 1 ex., G. Guzmán, 20 VII 1974, 1 ex., R. Granados, 16 XII 1974, 1 ex., L. Torres, 2 XI 1981, 1 ex., C. Orjuela & E. Mejia; **Tolima**, Espinal, 322 m, 4°09'N/74°53'W, 11 VI 1975, 1 ex., D. Martinez; **Tolima**, Fresno, 1473 m, 5°09'16"N/75°02'23"W, 19 II 1967, 1 ex., A. Moreno; **Tolima**, Honda, 224 m, 5°12'25"N/74°44'28"W, 5 I 1975, 1 ex., 4 I 1995, 1 ex., R. Granados; **Valle del Cauca**, Cali, 987 m, 3°26'N/76°31'W, 11 XI 1974, 1 ex., A. Contreras.

**Distribution.** Brazil; Colombia; Ecuador; Peru; Venezuela.

***Discomorpha spectanda* (Boheman, 1862)**

**Magdalena**, Mompox, 9°21'44"N/79°59'8"W, IX 1982, 1 ex. on *Tabebuia rosea*, A. Madrigal.

**Note.** The species is not associated with *Tabebuia rosea* and was most likely just sitting on the plant as *Discomorpha* is associated with Boraginaceae, dominantly the genus *Cordia* (L. Sekerka pers. comm.).

**Distribution.** Known only from Colombia.

***Discomorpha panamensis* (Spaeth, 1919b)**

**Antioquia**, San Luis, 1050 m, 6°02'50"N/74°59'59"W, 25 IX 1989, 1 ex., C. Rincón.

**Distribution.** Panama. **New to Colombia**.

***Dorynota kiesenwetteri* (Boheman, 1854)**

**Meta**, Villavicencio, 467 m, 4°09'N/73°39'W, 1 XI 1974, 1 ex., A. Yañez.

**Distribution.** Bolivia; Brazil; Colombia; Peru.

***Dorynota nodosa* (Boheman, 1854)**

**Sucre**, El Piña, 237 m, 9°27'N/75°12'W, 10 VII 1998, 1 ex., E. Vergara.

**Distribution.** Colombia; Panama; Venezuela.

***Dorynota rufomarginata* (Wagener, 1881)**

**Meta**, Villavicencio, 467 m, 4°09'N/73°39'W, 11 VII 1974, 1 ex., J. Pabón.

**Distribution.** Brazil. **New to Colombia**.

***Echoma anaglyptoides* Borowiec, 1998b**

**Cundinamarca**, Bituima, 1412 m, 4°52'31"N/74°32'33"W, 16 V 1989, 1 ex., M. Buitrago, 2 V 1992, 1 ex., O. Bilvao; **Cundinamarca**, Nimaima, Inspección de Policia de Tobia, 1185 m, 5°07'44"N/74°23'20"W, 10 I 1990, 1 ex., J. Garcia; **Cundinamarca**, Pacho, 1798 m, 5°07'N/74°09"W, 4 XI 1989, 1 ex, en Melastomataceae, C. Berrio; **Tolima**, Chaparral, La Granja, Barrio Baltrán, 850 m, 3°43'N/75°29"W, 5 IV 2002, 2 exx., M. Segura; **Tolima**, Espinal, 322 m, 4°09'10"N/74°53'19"W, 3 VI 1989, 1 ex., J. Rodriguez.

**Distribution.** Brazil; Colombia; French Guyana.

***Echoma clypeata* (Panzer, 1798)**

**Meta**, Villavicencio, 467 m, 4°09'N/73°39'W, 27 IX 1974, 1 ex., S. Vega.

**Distribution.** Bolivia; Brazil; Colombia; Ecuador; French Guyana; Guyana; Paraguay; Peru; Trinidad; Venezuela.

***Eugenysa columbiana* (Boheman, 1850)**

**Boyacá**, Otanche, 1050 m, 5°49'35"N/74°11'20’’, V 1994, 1 ex, A. Ortega; **Caldas**, Confines, Cariaño, 500 m, II 2002, 1 ex., E.E. Martinez; W **Cundinamarca**, Viani, 1498 m, 4°52'36"N/74°33'57"W, 24 IX 1985, 1 ex., Moro.

**Distribution.** Colombia; Costa Rica; Panama.

***Eugenysa martae* Borowiec, 1987**

**Valle del Cauca**, Calima, R. [= Río] Chancos, X 1990, 1 ex., L.C. Pardo-Locamo.

**Distribution.** known only from Colombia.

***Eugenysa unicolor* Borowiec & Dąbrowska, 1997**

**Cesar**, Aguachica, 162 m, 8°18'42"N/73°27'03"W, 10 VI 1972, 1 ex., J. Jiménez.

**Distribution.** Eucador; Peru; **New to Colombia**.

***Eurypedus nigrosignatus* (Boheman, 1854)**

**Atlántico**, Barranquilla, 68 m, 11°00'N/74°48'W, 7 VII 1967, 1 ex., Laverde; **Caldas**, Victoria, 700 m, 5°19'N/74°54'W, 2 IX 1974, 1 ex., M. Calderón; **Casanare**, Yopal, La Nieta, Maracaito, 350 m, 5°21'N/72°24'W, 16 IX 2003, 1 ex., J. López; **Casanare**, Yopal, Via La Chaparrera, 350 m, 5°21'N/72°24'W, 25 VIII 1997, 1 ex., H. Alvarez; **Cundinamarca**, Apulo, 421 m, 4°31’’/74°35'W, 5 X 1969, 1 ex., C. Pinzón, 24 IV 1993, 1 ex., I. Guarinmelgar; **Cundinamarca**, Bogotá, 2600 m, 4°35'56"N/74°04'51"W, 21 X 1966, 1 ex., J. Ortiz; **Cundinamarca**, Bogotá, Parque Simón Bolivar, 2620 m, 4°35'56"N/74°04'51"W, 20 XI 2001, 1 ex., W. Cañón; **Cundinamarca**, Choachi, 1927 m, 4°31'53"N/73°55'36"W, 20 III 2001, 1 ex., J. Valderama; **Cundinamarca**, Girardot, 281 m, 4°18'18"N/74°48'08"W, 15 V 1975, 1 ex., B. Guzman, 150 m, 4°30'N/75°45'W, 5 XI 1994, 1 ex., M. Beltrán, 24 XI 2001, 1 ex., D. Moreno; **Cundinamarca**, Guayabetal de Siquima, 1630 m, 4°52'N/74°28'W, 24 V 1964, 1 ex., N. Correa, 26 X 1969, 1 ex., C. Pinzón; **Cundinamarca**, La Mesa, Vda. San Joaquin, 670 m, 4°38'05"N/74°27'57"W, 17 IV 2002, 1 ex., J. Gómez, 17 IV 2002, 1 ex., A. Bejarano, 17 IV 2002, 1 ex., J. Cárdenas, 1700 m, 17 IV 2002, 1 ex., H. Duque; **Cundinamarca**, La Mesa, Via Tocaima, 1298 m, 4°38'N/74°27'W, 3 IV 1993, 1 ex., C. Omar; **Cundinamarca**, Nariño, 400 m, 4°24'N/74°50'W, 27 III 1975, 1 ex., A. Mendoza; **Cundinamarca**, Pacho, 1798 m, 4°18'18"N/74°48'08"W, 2 V 1998, 1 ex., Amorocho & Gómez; **Cundinamarca**, Pacho, Los Algarrobos, 1798 m, 5°07'N/74°09'W, 21 X 1997, 1 ex., J. Gross; **Cundinamarca**, Tocaima, 400 m, 4°27'40"N/74°38'10"W, 15 I 1961, 1 ex., A. Enciso, 24 IV 1969, 3 exx., L. Rico & C. Cujia, 28 III 1984, 2 exx., L. Sarmiento; **Cundinamarca**, Útica, 497 m, 5°11'45"N/74°29'03"W, 2 IV 1966, 3 exx., 3 IV 1966, 2 exx., J. Ortiz; **Cundinamarca**, Villeta, 842 m, 5°00'N/74°28'W, 17 VII 1966, 1 ex., E. Aponte, 18 X 1969, 1 ex., C. Pinzón; **Huila**, El Hobo, 276 m, 2°35'17"N/75°27'13"W, 11 V 1998, 1 ex., Amorocho & Gómez; **Huila**, Neiva, 442 m, 2°55'N/75°17'W, 13 IV 1964, 1 ex., F. Ramos, 25 V 1974, 5 exx., C. Reyes; **Meta**, Acacias, 522 m, 4°00'N/73°46'W, 13 X 1973, 1 ex., B. Torres; **Norte de Santander**, Cúcuta, 320 m, 7°53'N/72°30'W, 3 IV 1969, 1 ex., 3 IV 1969, 1 ex., 4 IV 1969, 1 ex., R. Lemus, 16 VII 1974, 1 ex., R. Granados, 20 VII 1974, 2 exx., J. Gómez, 9 X 1974, 1 ex., C. Melo; **Tolima**, Espinal, 322 m, 4°09'10"N/74°53'19"W, 19 V 1966, 1 ex., H. Ayala, 1 III 1967, 1 ex., Bernal; **Tolima**, Espinal, Ins. Pol. Chicoral, 322 m, 4°09'N/74°53'W, 23 XII 1969, 1 ex., J.D. Moreno; **Tolima**, Guamo, 200 m, 3°45'N/76°54'W, 8 XI 1996, 1 ex., K.R., 8 XII 2001, 1 ex., D. Moreno; **Tolima**, Honda, 229 m, 5°12'25"N/74°44'28"W, 8 V 1966, 1 ex., E. Guzmán, 1 ex., A. González; **Tolima**, Mariquita, 328 m, 5°12'10"N/74°55'49"W, 24 VII 1966, 1 ex, G. Pedraza, 22 IX 1993, 1 ex., P. Bolivar; **Tolima**, Melgar, 323 m, 4°12'N/74°38'W, 20 VII 1966, 1 ex, H. Santos, 1 V 1967, 1 ex., G. Torrado, 1 X 1994, 1 ex., W. Valera.

**Distribution.** Colombia; Guatemala; Nicaragua; Panama; Venezuela.

***Helocassis crucipennis* (Boheman, 1855)**

**Sucre**, San Marcos, Santa Inés, 29 m, 8°40'N/75°08'W, 26 VI 2003, 1 ex., A. Diaz.

**Distribution.** Belize; Colombia; Costa Rica; Guatemala; Honduras; Mexico; Nicaragua; Panama; Venezuela.

***Helocassis testudinaria* (Boheman, 1855)**

**Antioquia**, Santafé de Antioquia1567 m, 6°11'N/75°34'W, XII 1986, 3 exx., R. Vélez; **Cundinamarca**, Ricaurte, 287 m, 4°16'38"N/74°46'41"W, 29 III 1994, 1 ex., H. Gómez; **Cundinamarca**, Villeta, Vda. Guamalotal, 842 m, 5°00'N/74°28'W, 25 IX 2000, 1 ex., Urrea & Tamara.

**Distribution.** Belize; Colombia; Costa Rica; El Salvador; Guatemala; Honduras; Mexico; Panama; USA: Arizona, Florida; Venezuela.

***Hilarocassis bordoni* Borowiec, 2002a**

**Cundinamarca**, Girardot, 150 m, 4°30'N/75°45'W, 9 IV 1994, 1 ex., A. Silva; **Cundinamarca**, Guayabe tal, 1200 m, 4°13'N/73°48'W, 1 IV 1969, 1 ex., C. Acosta; **Cundinamarca**, La Mesa, 1298 m, 4°38'05"N/74°27'57"W, 15 IX 1996, 1 ex., C. Pinzón; **Cundinamarca**, Mesitas del Colegio, Fca. Las Brisas, 1100 m, 4°35'N/74°26'W, 9 III 2002, 1 ex., A. Bejarano.

**Distribution.** Venezuela. **New to Colombia**.

***Hybosa galbanata* Boheman, 1855**

**Caldas**, La Dorada, 178 m, 5°27'N/74°40'W, 10 V 1971, 1 ex., 15 X 1971, 1 ex., J. Zapata.

**Distribution.** known only from Colombia.

***Ischnocodia annulus* (Fabricius, 1781)**

**Cundinamarca**, Bogotá, El Paraiso, 2620 m, 4°35'56"N/74°04'51"W, 9 IX 1997, 1 ex., M. Alape; **Cundinamarca**, Cachipay, Insp. Pol. Anolaima, Vrda. San Cayetano, 5°16'22"N/74°34'22"W, 23 VII 1997, 1 ex., S. Olarte; **Cundinamarca**, Caqueza, 1746 m, 4°24'30"N/73°56'58"W, 12 IV 1997, 1 ex., A. Alessandri; **Cundinamarca**, La Mesa, Casco urbano, 1298 m, 4°38'05"N/74°27'57"W, 11 X 1997, 1 ex., E. Baron; **Cundinamarca**, La Mesa, San Javier, 1110 m, 4°38'N/74°27'W, 8 VI 1996, 1 ex., S. Dinas; **Cundinamarca**, La Mesa, Vrda. Guayabal, 1298 m, 4°38'05"N/74°27'57"W, 7 X 1997, 2 exx., M.A. & D.R.; **Cundinamarca**, Paime, 960 m, 5°22'16"N/74°09'18"W, 2 V 1992, 1 ex., Y. Castro; **Cundinamarca**, San Juan de Rio Seco, 1203 m, 4°50'54"N/74°37'35"W, 19 VIII 1998, 1 ex., J. Gutiérrez; **Cundinamarca**, Ubalá, Vrda. El Puerto, 1962 m, 4°44'48"N/73°32'18"W, 20 I 1998, 2 exx, M. Garcia; **Cundinamarca**, Villeta, Ecopetrol, 842 m, 5°00'52"N/74°28'23"W, 31 VIII 1997, 1 ex., R. Paredes; **Meta**, Cubarral, San Luis de Cubarral, Brisas del Ariari, 180 m, 3°47'N/73°52'W, 6 IV 2004, 1 ex., L. Ramirez & A. Vargas; **Meta**, La Macarena, 3°11'16"N/73°59'20"W, 26 III 1997, 1 ex., E. Bastidas; **Norte de Santander**, El Zulia, Rio Zulia, 220 m, 7°56'04"N/72°16'37"W, 2 XII 1998, 1 ex., F. Camacho; **Santander**, El Carmen de Chucuri, Vda. Dos Bocas, Fca. Playa Grande, 550 m, 6°46.514'N/73°38.408'W, XI 2003, 7 exx., L. Otero; **Santander**, La Belleza, Vrda. Los Naranjos, 1900 m, 5°51'57"N/73°58'02"W, 21 III 1997, 1 ex., H. Marin; **Valle del Cauca**, Buga, 969 m, 3°54'06"N/76°18'14"W, 14 X 2003, 1 ex., D. Quintana.

**Distribution.** Argentina; Belize; Bolivia; Brazil; Colombia; Costa Rica; Ecuador; El Salvador; French Guyana; Guatemala; Honduras; Mexico; Nicaragua; Panama; Paraguay; Trinidad; Venezuela.

***Microctenochira aspersa* (Champion, 1894)**

**Antioquia**, Cocorná, 6°3'25"N 75°11'7"W, 1286 m, VI 1977, 1 ex., R. Vélez; **Antioquia**, Concepción, 1862 m, 6°23'55"N/75°15'22"W, II 1997, 4 exx., F.J. Serna & J.G. Hurtado; **Cundinamarca**, Girardot, 150 m, 4°30'N/75°45'W, 13 VI 1967, 1 ex., Chavarriaga; **Cundinamarca**, Guayabetal, 1200 m, 4°13'N/73°48'W, 1 V 1969, 1 ex., L.M. Rico & C. Cujia; **Cundinamarca**, Sasaima, 1225 m, 4°57'59"N/76°26'15"W, 1 V 1970, 1 ex., E. Morales, VI, 1 ex., Jiménez; **Cundinamarca**, Viotá, 567 m, 4°26'31"N/74°31'33"W, 1 V 1996, 1 ex., L. Sánchez; **Magdalena**, Sevilla, VIII 1942, 2 exx., F.L. Gallego; **Meta**, Cubarral, San Luis de Cubarral, Brisas del Ariari, 180 m, 3°47'N/73°52'W, 6 IV 2004, 1 ex., L. Ramirez & A. Vargas; **Meta**, Villavicencio, 467 m, 4°09'N/73°39'W, 28 IV 1989, 1 ex., J. Rodriguez; **Santander**, Lebrija, 1015 m, 7°06'59"N 73°13'13"W, 12 V 1977, 1 ex., L. Rivera.

**Distribution.** Colombia; Costa Rica; Guatemala; Mexico; Panama.

***Microctenochira cumulata* (Boheman, 1855)**

**Antioquia**, San Andrés, 1530 m, VII 1952, 1 ex., F. Gallego.

**Distribution.** Colombia; Costa Rica; Ecuador; Guatemala; Mexico; Nicaragua; Panama; Venezuela.

***Microctenochira fairmairei* (Boheman, 1855)**

**Antioquia**, Amalfi, Cañón del Porce, Fosforito, 945 m, 6°45'37"N/75°06'28"W, 10 VI 1997, 2 exx., J. Hurtado; **Antioquia**, Amalfi, Cañón del Porce, Santa Lucia, 1050 m, 6°46'34"N/75°06'18"W, 31 VII 1997, 1 ex., J. Hurtado; **Antioquia**, San Roque, Vda. La Mora, Alto El Cuatro, 1800 m, 6°29'45"N/75°01'54"W, X 2008, 1 ex., H. Paredes.

**Distribution.** Bolivia; Colombia; Ecuador; Panama; Peru.

***Microctenochira flavonotata* (Boheman, 1855)**

**Cundinamarca**, La Mesa, 1298 m, 4°38'05"N/74°27'57"W, 11 VI 1989, 1 ex., A. Boada.

**Distribution.** Colombia; Costa Rica; Honduras; Nicaragua; Panama; Surinam; Trinidad; Venezuela.

***Microctenochira fraterna* (Boheman, 1855)**

**Antioquia**, Amalfi, Cañón del Porce, 6°55'N/75°04'W, 1050 m, 1997, 1 ex., J. Hurtado; **Antioquia**, La Estrella, 1764 m, 6°09'N/75°39'W, VI 1963, 1 ex., F.L. Gallego; **Antioquia**, Santafé de **Antioquia**, 550 m, 6°33'N/75°49'W, III 1998, 1 ex., J.E. Jaramillo; **Cundinamarca**, San Cayetano, 2208 m, 5°18"N/74°04'W, 3 X 1970, 2 exx., A. Tobón; **Cundinamarca**, Tena, 1384 m, 4°39'33"N/74°23'28"W, 8 XII 1995, 1 ex., Porras; **Cundinamarca**, Viotá, 567 m, 4°26'31"N/74°31'33"W, 9 IV 1999, 1 ex., V. Contreras.

**Distribution.** Colombia; Costa Rica; Ecuador; Nicaragua; Panama; Trinidad and Tobago; Venezuela.

***Microctenochira jousselini* (Boheman, 1855)**

**Boyacá**, Maripi, Vrda. Sánta Rosa, 800 m, 5°33'08"N/74°01'00"W, 12 IX 1999, 1 ex., C. Cortés.

**Distribution.** Colombia; Trinidad.

***Microctenochira lindigi* (Kirsch, 1865)**

**Antioquia**, La Unión, 2479 m, 5°58'N/75°21'W, 18 III 1998, 1 ex., J.E. Jaramillo; **Cundinamarca**, Medina, 431 m, 4°38'54"N/73°19'37"W, 16 X 1999, 1 ex., M. Acosta.

**Distribution.** Colombia; Bolivia; Ecuador; Venezuela.

***Microctenochira peltata* (Boheman, 1855)**

**Cundinamarca**, Sasaima, 1225 m, 4°57'59"N/76°26'15"W, 10 VI 1972, 1 ex., R. Arenas.

**Distribution.** Bolivia; Brazil; Ecuador; Peru. **New to Colombia**.

***Microctenochira quadrata* (DeGeer, 1775)**

**Antioquia**, Anori, 1574 m, 7°04'N/75°09'W, 5 IV 1985, 1 ex., R.R.D; **Cundinamarca**, Guayabetal, 1200 m, 4°13'N/73°48'W, 15 III 1969, 1 ex., A. Avila; **Cundinamarca**, Villeta, 842 m, 5°00'52"N/74°28'23"W, 26 V 1979, 1 ex., O. Garzón; **Meta**, Granada, 450 m, 3°32'N/73°43'W, 17 VII 1979, 1 ex., Gutierrez.

**Distribution.** Brazil; Colombia; French Guyana; Guyana; Panama; Paraguay; Surinam; Trinidad; Venezuela.

***Microctenochira semilobata* (Wagener, 1877)**

**Antioquia**, La Pintada, 678 m, 5°44'N/75°35'W, X 1971, 1 ex., R. Vélez.

**Distribution.** Brazil; Colombia.

***Microctenochira semilunaris* (Boheman, 1862)**

**Boyacá**, San Luis de Gaceno, 630 m, 4°49'21"N/73°10'13"W, 17 V 1972, 1 ex., S. Cubides; **Cundinamarca**, Arbelaez, 1417 m, 4°16'37"N/74°24'35"W, 13 VIII 1994, 1 ex., N. Rocio; **Cundinamarca**, Medina, Finca Mi Negro, 431 m, 4°38'54"N/73°19'37"W, 16 X 1999, 1 ex., M. Acosta; **Cundinamarca**, Guayabetal, 1200 m, 4°13'40"N/73°48'59"W, 12 III 1970, 1 ex., M. Garcia; **Cundinamarca**, Guayabetal de Siquima, 1630 m, 4°52'N/74°28'W, 15 VIII 1968, 1 ex., E. Gil; **Cundinamarca**, Sasaima, 1225 m, 4°57'59"N/76°26'15W, 26 V 1967, 1 ex., E. Delgado; **Cundinamarca**, Silvania, 1470 m, 4°24'21"N/74°23'24"W, 7 IV 1978, 1 ex., A. Sánchez; **Cundinamarca**, Soacha, 2568 m, 4°35'N/74°13'W, 26 IV 1975, 1 ex., R. Herrera; **Cundinamarca**, Villeta, 842 m, 5°00'N/74°28'W, 1 X 1970, 1 ex., G. Garcia; **Meta**, Villavicencio, 467 m, 4°09'N/73°39'W, IV 1969, 1 ex., Preciado, 19 IV 1969, 1 ex., L. Castiblanco.

**Distribution.** Bolivia; Brazil; Colombia; Ecuador; French Guyana; Peru.

***Microctenochira sepulchlaris* (Boheman, 1855)**

**Antioquia**, **Caldas**, 1768 m, 6°05'N/75°38'W, XI 1973, 1 ex., A. Madrigal; **Antioquia**, Concepción, 1862 m, 6°23'55"N/75°15'22"W, II 1997, 1 ex., F.J. Serna & J.G. Hurtado.

**Distribution.** Colombia.

***Microctenochira sertata* (Erichson, 1847)**

**Antioquia**, Chigorodó, 34 m, 7°40'13"N/76°41'00"W, VIII 1975, 1 ex., D. Gonzalez; **Antioquia**, Concepción, 1862 m, 6°23'55"N/75°15'22"W, II 1997, 1 ex., F.J. Serna & J.G. Hurtado; **Bolivar**, Monpós, **Córdoba**, 33 m, 9°14'00"N/74°26'00"W, XII 1994, 1 ex., J.A. Quiróz; **Córdoba**, Tierra alta, P.N.N. Nudo Paramillo, Cerro Murrucucu, 287 m, 7°59'24.27"N/76°07'44.29"W, IX-X 2004, 1 ex. J.E. Arango; **Cundinamarca**, Guayabetal, 1200 m, 4°13'40"N/73°48'59"W, 30 III 1972, 1 ex., R. Gómez; **Cundinamarca**, Sasaima, 1225 m, 4°57'59"N/76°26'15"W, IV 1977, 1 ex., Jaramillo; **Cundinamarca**, Villeta, 842 m, 5°00'52"N/74°28'23"W, 3 VI 1972, 1 ex., L. Barbosa; **Cundinamarca**, Viotá, 567 m, 4°26'31"N/74°31'33"W, 1 V 1996, 1 ex., L. Sánchez; **Meta**, Acacias, 522 m, 4°00'N/73°46'W, 1 V 1974, 1 ex., I. Oviédo.

**Distribution.** Bolivia; Brazil; Colombia; Ecuador; French Guyana; Peru; Surinam; Venezuela.

***Omaspides bistriata* Boheman, 1862**

**Valle del Cauca**, B. Dagua-Esacalarete, VI 1990, 1 ex., L.C. Pardo-Locamo.

**Distribution.** Colombia; Costa Rica; Panama; Venezuela.

***Omaspides nitidicollis* Spaeth, 1937a**

**Caldas**, La Dorada, 178 m, 5°27'24"N/74°40'02"W, V 1994, 1 ex., M. Garcia

**Distribution.** Colombia.

***Omaspides specularis* (Erichson, 1847)**

**Meta**, San Martin, 419 m, 3°42'N/73°42'W, 29 IV 1989, 1 ex., C. Rincón.

**Distribution.** Brazil; Colombia; Ecuador; Peru.

***Parachirida semiannulata* (Boheman, 1855)**

**Cundinamarca**, Villeta, 842 m, 5°00'52"N/74°28'23"W, 28 II 1980, 1 ex., Cifuentes.

**Distribution.** Brazil; Colombia; Peru.

***Parachirida subirrorata* (Boheman, 1855)**

**Córdoba**, Monteria, Tres Palmas, 18 m, 8°29'N/75°56'W, VII 1976, 1 ex., A. Madrigal; **Meta**, Cumaral, 480 m, 4°17'N/73°33'W, 16 IV 1989, 1 ex., C. Caballero; **Meta**, Villavicencio, 400 m, 4°09'N/73°39'W, 8 XI 2002, 1 ex., G. González; **Meta**, Villavicencio, Jardin Botánico, 400 m, 4°09'N/73°39'W, 8 XI 2002, 1 ex.

**Distribution.** Colombia; Costa Rica; Ecuador; Panama; Trinidad; Venezuela.

***Physonota alutacea* Boheman, 1854**

**Cundinamarca**, Girardot, 150 m, 4°30'N/75°45'W, 24 XI 2001, 1 ex., D. Moreno; **Cundinamarca**, Guaduas, 1007 m, 5°04'12"N/74°35'52"W, 6 V 1999, 1 ex., O. Higuera & R. Quevedo; **Cundinamarca**, San Francisco, 1570 m, 4°58'N/74°17'W, 1 VII 1970, 1 ex. ab. cyrtodes Boh., A. Moreno; **Cundinamarca**, Tocaima, 400 m, 4°27'40"N/74°38'18"W, 17 V 1969, 1 ex., A. Guzmán, 11 VII 1972, 1 ex., W. Toncel, 28 III 1994, 1 ex., L. Sarmiento; **Guajira**, Riohacha, 47 m, 11°33'N/72°554'W, 16 X|II 1974, 1 ex., A. Alarcón; **Meta**, Villavicencio, 467 m, 4°09'N/73°39'W, 18 XI 1994, 1 ex., G. González; **Sucre**, San Marcos, Santa Inés, 29 m, 8°40'N/75°08'W, 26 VI 2003, 1 ex. ab. cyrtodes Boh., A. Diaz; **Sucre**, Sincelejo, 200 m, 9°18'N/75°24'W, 12 V 1972, 1 ex., J. Vargas; **Tolima**, Melgar, 323 m, 4°12'24"N/74°38'44"W, 6 VI 1999, 2 exx., J. Arbelaez & F. Betancourt; **Tolima**, Purificación, 310 m, 3°51'N/74°56'W, 1 VI 1972, 2 exx., R. Arenas.

**Distribution.** Colombia; Costa Rica; Ecuador; Guatemala; Honduras; Mexico; Nicaragua; Panama; Trinidad; Venezuela; USA: Texas.

***Physonota pellucida* Wagener, 1877**

**Antioquia**, Jericó, 1967 m, 5°47'39"N/75°47'23"W, 1996, 8 exx., C. Tamayo; **Cundinamarca**, Caqueza, 1746 m, 4°24'30"N/73°56'58"W, 5 XI 1994, 1 ex., T. Luis.

**Distribution.** Colombia; Costa Rica; Nicaragua.

***Plagiometriona boschmai* Spaeth, 1937b**

**Antioquia**, Andes, Farallones del Citará, 1780 m, 5°45'33"N/76°03'42"W, 13 III 1999, 1 ex., D. Betancur; **Antioquia**, Andes, vereda La Siria, 2100 m, 24 IX 2000, 2 exx., S. Gomez; **Antioquia**, Andes, vereda Quebrada, Arriba, La Siria, 2200 m, 14 III 1999, 1 ex.; **Antioquia**, Frontino, 1317 m, 6°47'02"N/76°07'53"W, VI 1990, 1 ex., G. Morales; **Antioquia**, Urrao, 1790 m, 6°18'56"N/76°07'58"W, VI 1982, 1 ex., R. Bernal.

**Distribution.** Bolivia; Colombia; Ecuador; Venezuela.

***Plagiometriona latemarginata* Borowiec, 2001**

**Antioquia**, Frontino, 1317 m, 6°46'N/76°08'W, VII 1989, 1 ex., G. Morales & C. Mantilla.

**Distribution.** Panama. **New to Colombia**.

***Plagiometriona pehlkei* Spaeth, 1912**

**Boyacá**, San Pedro de Iguaque, 2750 m, 5°38'N/73°31'W, 7 V 1988, 1 ex., Lara; **Boyacá**, Villa de Leyva, Vda. Capilla, 2143 m, 5°38'N/73°31'W, 2 VI 2001, 1 ex., V. Quintero; **Cundinamarca**, Gachetá, “La Cima”, Fca. Bellavista, 1716 m, 4°55'N/73°51'W, 16 XI 2003, 1 ex., J. Lozano; **Cundinamarca**, La Mesa, 1298 m, 4°38'05"N/74°27'57"W, 14 III 1981, 1 ex., León & Garcia; **Cundinamarca**, La Mesa, Laguna Pedro Palo, 1298 m, 4°38'05"N/74°27'57"W, 2 XI 1996, 1 ex., A. Romero, 15 II 1997, 1 ex., P. Acosta, 16 XI 1997, 1 ex., D. Useche; **Cundinamarca**, La Vega, 1215 m, 4°59'57"N/74°20'23"W, 29 III 1994, 1 ex., D. Moro; **Cundinamarca**, La Vega, Vrda. San Francisco, 1215 m, 4°59'57"N/74°20'23"W, 28 IV 1999, 1 ex., D. Reynales; **Cundinamarca**, San Antonio del Tequendama, 1521 m, 4°37'04"N/74°21'15"W, 12 X 1998, 1 ex., G. Castañeda; **Cundinamarca**, Tena, 1384 m, 4°39'33"N/74°23'28"W, 1 ex., A. Tovar; **Cundinamarca**, Tena, El Ospicio, 1384 m, 4°39'33"N/74°23'28"W, 22 XI 1997, 1 ex., J. Martinez & D. Vanegas; **Cundinamarca**, Tibirita, 1980 m, 5°03'00"N/73°10'31"W, 22 XI 2003, 1 ex., O. Munar; **Cundinamarca**, Une, Puente Piedra Rosa, 2420 m, 4°24'N/74°02'W, 20 V 2001, 1 ex., D. Torres; **Huila**, Iquira, Potrerito, Finca La Victoria, 1123 m, 2°39'07"N/75°38'23"W, 29 VIII 2003, 1 ex., L. Martinez; **Meta**, Villavicencio, 467 m, 4°09'N/73°39'W, 1 V 1995, 1 ex., E. Losano, 4 XI 2001, 1 ex., M. Guillén.

**Distribution.** Colombia; Venezuela.

***Plagiometriona perroudi* (Boheman, 1862)**

**Boyacá**, Villa de Leyva, Vda. Capilla, 5°38'N/73°31'W, 2143 m, 10 V 2001, 1 ex., V. Quintero.

**Distribution.** Colombia.

***Polychalca perforata* (Boheman, 1850)**

**Antioquia**, Col. Bosque, 2150 m, 6°59'N/75°57'W, 22 X 2007, 1 ex., D.J. Restrepo.

**Distribution.** Colombia.

***Polychalma multicava* (Latreille, 1811)**

**Antioquia**, Amalfi, Cañón del Porce, Calandria, 985 m, 6°46'49.61"N/75°05'53.1"W, 25 VI 1997, 1 ex., J.G. Hurtado; **Antioquia**, Amalfi, Cañón del Porce, Normandia, 1000 m, 6°46'15.9"N/75°06'11"W, II 1998, 1 ex., J.G. Hurtado; **Caldas**, La Dorada, 178 m, 5°27'24"N/74°40'02"W, 1 IV 1967, 1 ex., Alcaraz; **Casanare**, Nunchia, Paz, 440 m, 5°38'N/72°11'W, 1 IX 1967, 1 ex., E. Cotes; **Cesar**, Aguachica, 162 m, 8°18'42"N/73°27'03"W, 24 III 1989, 1 ex., M. Jiménez; **Cundinamarca**, Villeta, 842 m, 5°00'52"N/74°28'23"W, 2 V 1967, 1 ex., I. Giraldo, 15 VI 1967, 1 ex., A. Reyes, 16 IX 1973, 1 ex., M. Arevalo; **Magdalena**, Ciénaga, Cgto. Sevilla, 12 m, 11°00'N/74°15'W, 15 VI 1971, 1 ex., A. Martinez; **Meta**, San Martin, 419 m, 3°42'N/73°42'W, 28 IV 1989, 1 ex., C. Castillo; **Norte de Santander**, Cúcuta, 320 m, 7°53'N/72°30'W, 5 V 1973, 1 ex, A. Porras; **Santander**, Bucaramanga, 958 m, 7°07'N/73°07'W, 2 XI 1973, 1 ex., Castellano; **Sucre**, San Marcos, Santa Inés, 29 m, 8°40'N/75°08'W, 26 VI 2003, 1 ex., A. Diaz; **Tolima**, Mariquita, 328 m, 5°12'N/74°55'W, 12 III 1967, 1 ex., H. Reyes, 19 II 1971, 3 exx., 20 II 1971, 2 exx., G. Hurtado.

**Distribution.** Colombia; Costa Rica; Ecuador; Panama; Peru; Venezuela.

***Stolas blanda* (Boheman, 1850)**

**Huila**, Garzón, 828 m, 2°12'N/75°38'W, 4 I 1972, 1 ex., F. Ramirez.

**Distribution.** Brazil; Colombia; Ecuador.

***Stolas ephippium* (Lichtenstein, 1796)**

**Santander**, Landazuri, 1600 m, 6°13'N/79°45'W, VI 1984, 1 ex., Luengas.

**Distribution.** Brazil; Colombia; Costa Rica; Ecuador; Guatemala; Guyana; Nicaragua; Panama; Surinam.

***Stolas haematites* (Lichtenstein, 1796)**

**Antioquia**, Amalfi, Cañón del Porce, 1050 m, 6°46'N/75°05'W, 1997, 2 exx., J.G. Hurtado; **Antioquia**, Barbosa, 1308 m, 6°26'N/75°20'W, IX 1983, 1 ex., Barreiro; **Antioquia**, La Estrella, 1764 m, 6°09'N/75°39'W, XI 1981, 1 ex., B. Múnera; **Antioquia**, La Pintada, 682 m, 5°44'N/75°35'W, X 1971, 1 ex., R. Vélez; **Cundinamarca**, Mesitas del Colegio, 4°35'14"N/74°26'58"W, 7 X 1989, 1 ex., M. Beltrán; **Norte de Santander**, El Carmen, 761 m, 8°30'36"N/73°27'11"W, 15 III 1994, 1 ex.

**Distribution.** Brazil; Colombia; French Guyana; Paraguay: Presidente Hayes; Venezuela.

***Stolas lebasii* (Boheman, 1850)**

**Valle del Cauca**, Cali, 987 m, 3°26'N/76°31'W, 11 XI 1974, 1 ex., A. Contreras.

**Distribution.** Belize; Colombia; Costa Rica; Guatemala; Honduras; Mexico; Nicaragua; Panama; Trinidad.

***Stolas stolida* (Spaeth, 1917)**

**Cacuetá**, Florencia, 480 m, 1°36'N/75°37'W, 12 VIII 1968, 1 ex., J. Bobadilla; **Cundinamarca**, Cachipay, 474 m, 5°16'22"N/74°34'22"W, 13 V 1977, 1 ex., E. de León; **Meta**, Acacias, 522 m, 4°00'N/73°46'W, 12 III 1977, 1 ex., A. Cubides; **Meta**, La Macarena, 3°11'16"N/73°59'20"W, 23 III 1997, 1 ex., F. Villarmil, 26 III 1997, 1 ex., E. Bastidas; **Meta**, Villavicencio, 467 m, 4°09'N/73°39'W, 10 V 1967, 1 ex., H. Ramos, 20 VI 1967, 1 ex., E. Peralta, VII 1967, 1 ex., P. Pérez.

**Distribution.** Colombia; Ecuador.

***Stolas tachiraensis* Borowiec, 2009b**

**Boyacá**, San Pedro de Iguaque, 2750 m, 5°38'N/73°31'W, 7 V 1988, 1 ex., G. Negret; **Cundinamarca**, Anolaima, 1726 m, 4°45'54"N/74°28'08"W, 15 IV 1992, 1 ex., I. Garcia; **Cundinamarca**, Anolaima, Carretera via a Anolaima, 1656 m, 4°45'N/74°28'W, 8 IV 1989, 1 ex., C. Garzón; **Cundinamarca**, Cachipay, Insp. Pol. Anolaima, 5°16'22"N/74°34'22"W, 30 VIII 1988, 1 ex., E. Rivera; **Cundinamarca**, Caqueza, 1746 m, 4°24'30"N/73°56'58"W, 5 XI 1994, 1 ex., T. Luis; **Cundinamarca**, Fusagasugá, 1746 m, 4°20'49"N/74°21'53"W, 15 III 1992, 1 ex., Ramos & Quiroga; **Cundinamarca**, Girardot, 281 m, 4°18'18"N/74°48'08"W, 28 XII 1985, 1 ex., Torres & Viña, 28 V 1992, 1 ex., L. Narvaez; **Cundinamarca**, Guayabal de Siquima, Vda. El Resguardo, 4°53'N/74°28'W, 1630 m, 2005, 1 ex., 1636 m, 2005, 1 ex., F. Cruz; **Cundinamarca**, **Cundinamarca**, La Mesa, 1298 m, 4°38'05"N/74°27'57"W, 24 V 1992, 1 ex., A. Ariza & L. Ferrucho, 4 II 1994, 1 ex., Ubaque, 1 IV 1997, 1 ex., J. Camargo; **Cundinamarca**, La Vega, 1230 m, 4°59'57"N/74°20'23"W, 2 IV 1967, 1 ex., Ardila; **Cundinamarca**, Mesitas del Colegio, San José, 983 m, 4°35'N/74°26'W, 16 III 1975, 1 ex., A. Martinez, 4 XII 2004, 1 ex., S. Cubillos; **Cundinamarca**, Quipile, 1444 m, 4°44'48"N/74°32'14"W, 4 V 1990, 1 ex., S. Suárez; **Cundinamarca**, Silvania, 1470 m, 4°24'21"N/74°23'24"W, 3 X 1996, 1 ex., S. Fuentes; **Cundinamarca**, Villeta, 804 m, 5°00'52"N/74°28'23"W, 3 VI 1967, 1 ex., M. Contreras; **Meta**, Humadea Insp., Pol. Guamal, 518 m, 3°51'N/73°45'W, 6 V 1999, 1 ex., O. Higuera & R. Quevedo; **Meta**, Villavicencio, 467 m, 4°09'N/73°39'W, 25 X 1988, 1 ex., P. León; **Santander**, Barbosa, Cite Las Delicias, 1600 m, 5°53'N/73°34'W, 28 VIII 2004, 1 ex., D. Mejia, 1 ex., E. Villarraga, 20 IX 2004, 1 ex., C. Soto; **Tolima**, Ibagué, 1285 m, 4°26'50"N/75°14'44"W, 1 ex., González & Nortua; **Tolima**, Mariquita, 328 m, 5°12'10"N/74°55'49"W, 14 X 1995, 1 ex., Carlos.

**Distribution.** Venezuela. **New to Colombia**.

***Trilaccodea tomentosa* (Boheman, 1850)**

**Antioquia**, Medellin, 1538 m, 6°13'N/75°34'W, IX 1944, 1 ex., F.L. Gallego; **Boyacá**, Duitama, 2530 m, 5°49'35"N/73°02'32"W, 15 IV 1996, 1 ex., S. Alfonso; **Boyacá**, Tibaná, 2090 m, 5°19’ 13"N/73°23'59"W, 20 IV 1996, 1 ex., 10 V 1996, 2 ex., B. Velandia; **Valle del Cauca**, Cali, 987 m, I. 1944, 1 ex., F. Gallego.

**Distribution.** Colombia; Venezuela.

### Checklist of tortoise beetles of Colombia

[endemic species and department data in bold, uncertain and imprecise location in normal and in square brackets; colour photos of species marked with an asterisk (*) are available on web page by Borowiec and Świętojańska (2014)]

#### Tribe Cassidini

*Agroiconota judaica (Fabricius, 1781) – **Antioquia**, **Bolivar**, **Boyacá**, **Cesar**, **Córdoba**, **Cundinamarca**, **Meta**, **Norte de Santander**, **Santander**, **Tolima**, **Valle del Cauca**

*Agroiconota propinqua (Boheman, 1855) – **Antioquia**, **Atlántico**, **Bolivar**, **Caldas**, **Casanare**, **Chocó**, **Cundinamarca**, **Magdalena**, **Meta**, **Tolima**

*Agroiconota sodalis Spaeth, 1936a – [Rio Magdalena]

*Aidoia nubilosa Boheman, 1855 – **Cundinamarca**

*Charidotella amicula (Spaeth, 1936a) – **Valle del Cauca**

*Charidotella balteata (Champion, 1894) – **Antioquia**

*Charidotella bifasciata (Linnaeus, 1758) – **Norte de Santander**

*Charidotella carnulenta (Erichson, 1847) – **Caqueta**, **Meta**

*Charidotella circumnotata (Boheman, 1862) – **Antioquia**, **Huila**, **Santander**

*Charidotella glaucovittata (Erichson, 1847) – **Cundinamarca**, **Meta**, **Valle del Cauca**

*Charidotella immaculata (Olivier, 1790) – **Cundinamarca**, **Huila**, **Meta**, **Norte de Santander**, **Tolima**

*Charidotella incorrupta (Boheman, 1855) – **Antioquia**, **Cundinamarca**, **Meta**, **Tolima**

*Charidotella liquida (Erichson, 1847) – **Cundinamarca**, **Meta**

*Charidotella moraguesi Borowiec, 2007b – **Tolima**

*Charidotella myops (Boheman, 1855) – [Colombia]

***Charidotella oblectabilis* (Spaeth, 1926a)** – [Colombia]

*Charidotella puella (Boheman, 1855) – **Antioquia**, **Bolivar**, **Boyacá**, **Cesar**, **Cundinamarca**, **Meta**, **Tolima**, **Valle del Cauca**

*Charidotella purpurea (Linnaeus, 1758) – [Colombia]

*Charidotella sexpunctata (Fabricius, 1781) – **Antioquia**, **Cundinamarca**, **Huila**, **Meta**, **Santander**, **Tolima**, **Valle del Cauca**

*Charidotella tuberculata (Fabricius, 1775) – **Magdalena**, **Meta**, **Tolima**

*Charidotella vinula (Boheman, 1855) – **Cundinamarca**, **Valle del Cauca**

*Charidotis aurofasciata (Erichson, 1847) – [Colombia]

*Charidotis bipartita (Boheman, 1855) – **Santander**

*Charidotis cincticula (Boheman, 1855) – **Cundinamarca**

***Charidotis discicollis* Boheman, 1855** – [Colombia]

*Charidotis exigua Boheman, 1855 – [Colombia]

*Charidotis furva Boheman, 1855 – **Norte de Santander**

***Charidotis languida* Spaeth, 1936b** – [Colombia]

*Charidotis luteola Boheman, 1855 – [Colombia]

*Charidotis vitreata (Perty, 1830) – **Antioquia**, **Boyacá**

*Chersinellina heteropunctata (Boheman, 1854) – **Córdoba**, **Cundinamarca**, **Magdalena**

Coptocycla sp. near rufonotata Sekerka & Windsor in litt. – **Antioquia, Magdalena**

*Coptocycla robusta Spaeth, 1936c – [Colombia]

*Cteisella centropunctata (Boheman, 1855) [Colombia]

*Cteisella divalis Spaeth, 1926b – **Cundinamarca**, **Tolima**

*Cyclocassis secunda Borowiec, 1998a – **Norte de Santander**

*Deloyala fuliginosa (Olivier, 1790) – **Córdoba**, **Valle del Cauca**

*Deloyala insubida (Boheman, 1855) – **Antioquia**, **Cundinamarca**, **Meta**, **Tolima**, **Valle del Cauca**

*Helocassis crucipennis (Boheman, 1855) – **Sucre**

*Helocassis testudinaria (Boheman, 1855) – **Antioquia**, **Cundinamarca**

*Hybosa galbanata Boheman, 1855 – **Caldas**

***Hybosa unicolor* Wagener, 1877** – [Colombia]

*Ischnocodia annulus (Fabricius, 1781) – **Cundinamarca**, **Meta**, **Norte de Santander**, **Santander**, **Tolima**, **Valle del Cauca**

*Metrionella erratica (Boheman, 1855) – [Colombia]

*Metrionella placans Spaeth, 1932 – [Colombia - Cachabé; now the locality is in Esmeralda Province in Ecuador close to Colombian border thus occurrence of this species in recent Colombia needs confirmation]

*Metrionella tumacoensis Borowiec, 2002b – **Nariño**

***Microctenochira arcana* (Spaeth, 1926b)** – [Colombia – Nova Granada]

*Microctenochira aspersa (Champion, 1894) – **Antioquia**, **Cundinamarca**, **Magdalena**, **Meta**, **Santander**

*Microctenochira bifenestrata (Boheman, 1855) – **Cundinamarca**

***Microctenochira bogotana* (Spaeth, 1926b)** – **Cundinamarca**

*Microctenochira cumulata (Boheman, 1855) – **Antioquia**

*Microctenochira diffinis (Boheman, 1855) – **Antioquia**, **Amazonas**, **Cundinamarca**

*Microctenochira fairmairei (Boheman, 1855) – **Antioquia**

*Microctenochira flavonotata (Boheman, 1855) – **Cundinamarca**

*Microctenochira fraterna (Boheman, 1855) – **Antioquia**, **Boyacá**, **Cundinamarca**, **Valle del Cauca**

***Microctenochira impolluta* (Spaeth, 1926b)** – **Antioquia**

*Microctenochira jousselini (Boheman, 1855) – **Boyacá**

*Microctenochira libidinosa (Spaeth, 1926b) – **Valle del Cauca**

*Microctenochira lindigi (Kirsch, 1865) – **Antioquia**, **Cundinamarca**, **Meta**

*Microctenochira lugubris (Boheman, 1862) – [Colombia]

*Microctenochira nigrocincta (Wagener, 1877) – **Bolivar**

*Microctenochira peltata (Boheman, 1855) – **Cundinamarca**

*Microctenochira porosa (Boheman, 1855) – **Cundinamarca**

*Microctenochira quadrata (DeGeer, 1775) – **Antioquia**, **Cundinamarca**, **Meta**

*Microctenochira reticularis (DeGeer, 1775) – **Antioquia**, **Cundinamarca**, **Putumayo**

*Microctenochira rubrocincta (Boheman, 1855) – [Colombia]

*Microctenochira semifasciata* (Boheman, 1855) – [Colombia]

*Microctenochira semilobata (Wagener, 1877) – **Antioquia**

*Microctenochira semilunaris (Boheman, 1862) – **Antioquia**, **Boyacá**, **Cundinamarca**, **Meta**

*Microctenochira sepulchralis (Boheman, 1855) – **Antioquia**, **Cundinamarca**

*Microctenochira sertata (Erichson, 1847) – **Antioquia**, **Bolivar**, **Boyacá**, **Córdoba**, **Cundinamarca**, **Meta**, **Putumayo**, **Valle del Cauca**

***Nuzonia ibaguensis* Spaeth, 1912** – **Tolima**

*Nuzonia marginepunctata Borowiec, 2000 – **Caldas**, **Valle del Cauca**

*Orexita blattoides Spaeth, 1911 – [Colombia]

*Orexita justini (Boheman, 1855) – **Cundinamarca**

*Orexita plagipennis Spaeth, 1911 – [Colombia]

*Orexita subgibbosa Spaeth, 1911 – **Valle del Cauca**

*Parachirida flavolineata (Latreille, 1811) – **Bolivar**, **Santander**

*Parachirida semiannulata (Boheman, 1855) – **Antioquia**, **Cundinamarca**

*Parachirida subirrorata (Boheman, 1855) – **Córdoba**, **Meta**

***Plagiometriona aucta* (Boheman, 1855)** – [Colombia]

***Plagiometriona bisbimaculata* (Boheman, 1855)** – [Colombia]

*Plagiometriona boschmai Spaeth, 1937b – **Antioquia**, **Cundinamarca**, **Tolima**, **Valle del Cauca**

*Plagiometriona buqueti (Boheman, 1855) – **Boyacá**, **Cundinamarca**, **Tolima**

***Plagiometriona columbica* Spaeth, 1937b** – [Colombia]

***Plagiometriona fragilicornis* Spaeth, 1937b** – **Tolima**

*Plagiometriona glyphica* (Boheman, 1855) – **Tolima**

*Plagiometriona latemarginata Borowiec, 2001 – **Antioquia**

***Plagiometriona nobilis* Spaeth, 1937b** – **Tolima**

*Plagiometriona pehlkei Spaeth, 1912 – **Boyacá**, **Cundinamarca**, **Huila**, **Meta**, **Tolima**

*Plagiometriona pernix Spaeth, 1912 – **Tolima**

*Plagiometriona perroudi (Boheman, 1862) – **Boyacá**, **Cauca, Cundinamarca**

*Plagiometriona phoebe (Boheman, 1855) – **Antioquia**, **Boyacá**

***Plagiometriona ramosa* (Boheman, 1855)** – [Colombia]

***Plagiometriona resplendens* (Kirsch, 1865)** – **Cundinamarca**

*Plagiometriona steinheili (Wagener, 1877) – **Norte de Santander**

*Plagiometriona zelleri (Boheman, 1855) – **Boyacá**, **Cundinamarca**

#### Tribe Delocraniini

*Delocrania cossyphoides Guérin, 1844 – **Magdalena**, **Santander**

#### Tribe Dorynotini

*Dorynota electa (Spaeth, 1923) – **Risaralda**

*Dorynota hastifera* (Spaeth, 1923) – [Colombia]

*Dorynota insidiosa (Boheman, 1854) – [Colombia]

*Dorynota kiesenwetteri (Boheman, 1854) – **Meta**

*Dorynota nodosa (Boheman, 1854) – **Sucre**

*Dorynota rufomarginata* (Wagener, 1881) – **Meta**

*Dorynota truncata (Fabricius, 1781) – [Colombia]

#### Tribe Eugenysini

*Agenysa connectens (Baly, 1869) – **Antioquia**, **Huila**

*Agenysa crassicornis Spaeth, 1905 – **Risaralda**, **Santander**

*Eugenysa columbiana (Boheman, 1850) – **Antioquia**, **Bolivar**, **Boyacá**, **Caldas**, **Cundinamarca**, **Valle del Cauca**

*Eugenysa martae Borowiec, 1987 – **Valle del Cauca**

*Eugenysa regalis (Boheman, 1850) – [Colombia]

*Eugenysa unicolor Borowiec & Dąbrowska, 1997 – **Cesar**

*Miocalaspis gentilis (Erichson, 1847) – [Colombia]

#### Tribe Goniocheniini

*Chlamydocassis bicornuta (Boheman, 1850) – [Colombia]

*Goniochenia buckleyi (Baly, 1872) – [Colombia]

*Goniochenia elocata (Boheman, 1850) – **Antioquia**, **Valle del Cauca**, **Tolima**

*Polychalma multicava (Latreille, 1811) – **Antioquia**, **Boyacá**, **Caldas**, **Casanare**, **Cesar**, **Cundinamarca**, **Magdalena**, **Meta**, **Norte de Santander**, **Sucre**, **Tolima**, **Valle del Cauca**

#### Tribe Hemisphaerotini

*Spaethiella circumdata (Boheman, 1850) – **Bolivar**, **Cundinamarca**

*Spaethiella coccinea (Boheman, 1850) – **Amazonas**, **Meta**

*Spaethiella flexuosa* (Champion, 1893) – **Magdalena**

*Spaethiella laevicollis (Spaeth, 1910) – **Tolima**

*Spaethiella miniata (Boheman, 1856) – [Colombia]

*Spaethiella pulchella (Baly, 1859) – [Colombia]

*Spaethiella purpureocincta* (Spaeth, 1929) – **Cundinamarca**

*Spaethiella quadrata (Spaeth, 1902) – **Caquetá**

*Spaethiella robusta (Spaeth, 1910) – **Norte de Santander**, **Tolima**, [Colombia – Villa Carolina, Villa Elvira]

*Spaethiella sanguinea (Fabricius, 1801) – [Colombia]

*Spaethiella sublaevis (Spaeth, 1901) – **Valle del Cauca**

*Spaethiella valida (Spaeth, 1901) – **Valle del Cauca**

Tribe Mesomphaliini

*Acromis sparsa (Boheman, 1854) – **Antioquia**, **Boyacá**, **Cundinamarca**, **Norte de Santander**, **Santander**, **Tolima**, **Valle del Cauca**

*Acromis spinifex (Linnaeus, 1763) – [Colombia]

*Acromis venosa (Erichson, 1847) – [Colombia]

*Botanochara ordinata (Boheman, 1850) – **Santander**

*Chelymorpha alternans Boheman, 1854 – **Nariño**

***Chelymorpha atomaria* Boheman, 1854** – [Colombia]

*Chelymorpha cavata Boheman, 1854 – **Antioquia**, **Cundinamarca**

*Chelymorpha cribraria (Fabricius, 1775) – **Antioquia**, **Boyacá**, **Valle del Cauca**

*Chelymorpha infirma Boheman, 1854 – **Huila**

*Chelymorpha marginata (Linnaeus, 1758) – **Cesar**, **Cundinamarca**, **Meta**

*Chelymorpha praetextata Boheman, 1854 – **Boyacá**

***Chelymorpha stygia* Boheman, 1862** – **Cundinamarca**

*Chelymorpha testaceomarginata Boheman, 1854 – **Boyacá**, **Cundinamarca**, **Huila**, **Meta**, **Norte de Santander**, **Tolima**, **Valle del Cauca**

*Chelymorpha variolosa (Olivier, 1790) – **Antioquia**

*Cyrtonota abrili Borowiec & Świętojańska sp. n. – **Antioquia, Caldas**

*Cyrtonota balyi (Kirsch, 1883) – **Putumayo**

*Cyrtonota bergeali Borowiec & Sassi, 1999 – **Valle del Cauca**

*Cyrtonota bugaensis Borowiec & Sassi, 1999 – **Valle del Cauca**

*Cyrtonota caudata (Boheman, 1850) – **Caldas**, **Cesar**, **Risaralda**, **Tolima**

*Cyrtonota compulsa (Spaeth, 1909) – **Tolima**

*Cyrtonota dissecta (Boheman, 1850) – **Cundinamarca**, **Meta**, **Risaralda**, **Tolima**

*Cyrtonota gibbera Borowiec, 1989 – **Cundinamarca**

*Cyrtonota goryi (Boheman, 1850) – **Meta**, **Tolima**

*Cyrtonota kolbei (Spaeth, 1907) – **Huila**

*Cyrtonota lurida (Spaeth, 1913) – [Colombia]

*Cyrtonota moderata (Spaeth, 1913) – **Caldas**, **Quindio**

*Cyrtonota pavens (Spaeth, 1913) – [Colombia]

*Cyrtonota pyramidata (Boheman, 1850) – [Colombia]

*Cyrtonota santanderensis Borowiec, 2009a – **Santander**

*Cyrtonota serinus (Erichson, 1847) – **Amazonas**, **Huila**

*Cyrtonota steinheili (Wagener, 1877) – **Cundinamarca**, **Toledo**

*Cyrtonota textilis (Boheman, 1850) – **Cundinamarca**, **Nariño**

*Cyrtonota tigrina (Boheman, 1850) – [Colombia]

*Cyrtonota timida Sassi, 2008 – **Nariño**

*Echoma anaglypta (Boheman, 1862) – [Colombia]

*Echoma anaglyptoides Borowiec, 1998b – **Antioquia**, **Cundinamarca**, **Tolima**, **Valle del Cauca**

*Echoma clypeata (Panzer, 1798) – **Antioquia**, **Meta**

*Echoma signata (Panzer, 1798) – **Antioquia**, **Cundinamarca**

*Hilarocassis bordoni Borowiec, 2002a – **Cundinamarca**

*Hilarocassis evanida (Boheman, 1850) – [Colombia; occurrence of this species in Colombia needs confirmation because with great probability based on misidentification with *Hilarocassis bordoni* Borowiec]

*Omaspides augusta Boheman, 1856 – [Colombia – Rio Tacana]

*Omaspides bistriata Boheman, 1862 – **Valle del Cauca**

***Omaspides limbipennis* Spaeth, 1922** – **Meta**

*Omaspides nitidicollis Spaeth, 1937a – **Antioquia**, **Caldas**, **Santander**, **Tolima**, **Valle del Cauca**

*Omaspides specularis (Erichson, 1847) – **Meta**, **Valle del Cauca**

*Paraselenis nupta (Boheman, 1854) – **Cundinamarca**

*Paraselenis tersa (Boheman, 1854) – [Colombia]

*Stolas bioculata (Boheman, 1850) – [Colombia]

*Stolas blanda (Boheman, 1850) – **Huila**, **Meta**, **Tolima**

*Stolas coerulescens (Boheman, 1850) – **Antioquia**

*Stolas decemguttata (Sturm, 1828) – [Colombia]

*Stolas discoides (Linnaeus, 1758) – [Colombia]

*Stolas ephippium (Lichtenstein, 1796) – **Antioquia**, **Santander**, **Valle del Cauca**

*Stolas excelsa (Spaeth, 1917) – **Valle del Cauca**

*Stolas extricata (Boheman, 1850) – **Bolivar**, **Valle del Cauca**

*Stolas haematites (Lichtenstein, 1796) – **Antioquia**, **Cundinamarca**, **Norte de Santander**

*Stolas inexculta (Boheman, 1862) – **Risaralda**

*Stolas lebasii (Boheman, 1850) – **Valle del Cauca**

*Stolas napoensis Borowiec, 1998a – **Risaralda**

*Stolas niobe (Spaeth, 1919a) – **Risaralda**

*Stolas pertusa (Boheman, 1850) – [Colombia]

*Stolas pleurosticha (Erichson, 1847) – **Valle del Cauca**

*Stolas puberula (Boheman, 1856) – **Huila**, **Risaralda**

*Stolas quinquefasciata (Wagener, 1877) – [Colombia]

*Stolas rubroreticulata (Boheman, 1856) – **Boyacá**, **Cundinamarca**, **Tolima**

*Stolas stolida (Spaeth, 1917) – **Cacuetá**, **Cundinamarca**, **Huila**, **Meta**

*Stolas tachiraensis Borowiec, 2009b – **Boyacá**, **Cundinamarca**, **Meta**, **Santander**, **Tolima**

*Trilaccodea excisa (Boheman, 1856) – [Colombia]

*Trilaccodea tomentosa (Boheman, 1850) – **Antioquia**, **Boyacá**, **Valle del Cauca**

*Zatrephina lineata (Fabricius, 1787) – [Colombia]

#### Tribe Omocerini

*Canistra osculatii Guérin, 1855 – **Antioquia**, **Huila**, **Risaralda**, **Tolima**

*Canistra varicosa Erichson, 1847 – **Valle del Cauca**

*Discomorpha amazona (Spaeth, 1940) – **Meta**, **Santander**

*Discomorpha batesi (Boheman, 1856) – **Antioquia**

*Discomorpha bernhaueri (Spaeth, 1909) – **Valle del Cauca**

*Discomorpha biplagiata (Guérin, 1844) – **Bolivar**, **Casanare**, **Cundinamarca**, **Huila**, **Tolima**, **Valle del Cauca**

***Discomorpha breiti* (Spaeth, 1907)** – [Colombia]

*Discomorpha conspersipennis (Boheman, 1862) – **Antioquia**, **Bolivar**, **Cundinamarca**

***Discomorpha garitana* (Spaeth, 1919b)** – **Norte de Santander**

*Discomorpha giganteensis Borowiec, 2006a – **Huila**

*Discomorpha instabilis (Baly, 1872) – **Cundinamarca**, **Huila**, **Tolima**

*Discomorpha languinosa (Boheman, 1850) – [Colombia]

*Discomorpha mandli (Spaeth, 1909) – **Huila**

*Discomorpha miniata (Boheman, 1850) – **Antioquia**, **Huila**, **Putumayo**

*Discomorpha nigropunctata (Boheman, 1850) – **Boyacá**, **Valle del Cauca**

***Discomorpha nigrosanguinea* (Wagener, 1877)** – **Tolima**

*Discomorpha nigrosparsa (Wagener, 1877) – **Caldas**, **Valle del Cauca**

*Discomorpha panamensis (Spaeth, 1919b) – **Antioquia**

*Discomorpha peruviana (Boheman, 1850) – **Meta**

*Discomorpha salvini (Baly, 1864) – [Colombia]

***Discomorpha skalitzkyi* (Spaeth, 1911)** – [Colombia]

*Discomorpha spectanda (Boheman, 1862) – **Magdalena**

*Discomorpha waehneri (Spaeth, 1940) – **Huila**, **Putumayo**

*Discomorpha wingelmuelleri (Spaeth, 1907) – [Colombia]

*Omocerus casta (Boheman, 1862) – [Colombia]

***Omocerus caucanus* (Spaeth, 1931)** – **Valle del Cauca**

*Omocerus creberrimus (Boheman, 1850) – **Antioquia**, **Cundinamarca**

*Omocerus reichei (Boheman, 1850) – [Colombia; occurrence of this species in Colombia needs confirmation because with great probability based on misidentification]

*Omocerus relucens* (Spaeth, 1931) – **Cordoba**

*Omocerus smaragdinus (Boheman, 1850) - **Meta**

*Omocerus taurus (Fabricius, 1787) – [Colombia]

*Omocerus viridicoeruleus* (Boheman, 1850) – [Colombia]

*Polychalca perforata (Boheman, 1850) – **Antioquia**, **Boyacá**, **Cundinamarca**

#### Tribe Physonotini

*Cistudinella foveolata Champion, 1894 – **Guaviare**

*Eurypedus nigrosignatus (Boheman, 1854) – **Atlántico**, **Caldas**, **Casanare**, **Cundinamarca**, **Huila**, **Magdalena**, **Meta**, **Norte de Santander**, **Tolima**

*Physonota alutacea Boheman, 1854 – **Cundinamarca**, **Guajira**, **Meta**, **Sucre**, **Tolima**

*Physonota lutarella Boheman, 1856 – **Boyacá**, **Valle del Cauca**, **Tolima**

*Physonota pellucida Wagener, 1877 – **Antioquia**, **Cundinamarca**

***Physonota plana* Boheman, 1854** – **Cundinamarca**

## Discussion

South America with approximately 2/3 of described world cassids is a centre of diversity of tortoise beetles (Borowiec 1999, Borowiec and Świętojańska 2014a). Until recently only three complete checklists of species supplemented by new faunistic materials were published for Neotropical countries: Ecuador (Borowiec 1998a), Trinidad and Tobago (Chaboo and Borowiec 2003) and French Guyana (Borowiec and Moragues 2005), including descriptions of several species new to science (193, 38 and 121 species of tortoise beetles were recorded respectively). The lists and catalogue cited above suggest that western Andean countries (from Colombia to Bolivia) might have several endemic species. Summarized data of distribution of Neotropical tortoise beetles in the web page by Borowiec and Świętojańska (2014b) show that, among the Andean countries, the richest is the fauna of Peru – 323 species, being 81 endemics (25.1%), followed by Bolivia – 254 species, with 42 endemics (16.5%), and Ecuador – 214 species, with 63 endemics (29.4%). The present contribution presents 238 species and 60 endemics (25.2%), but we anticipate many more species since Colombia is much larger than Ecuador yet the two countries have similar numbers of recorded species. Out of the 238 listed species recorded from Colombia 62 (26.1%) still have imprecise location.

## Supplementary Material

XML Treatment for Cyrtonota abrili
